# Tumour‐associated macrophage‐derived DOCK7‐enriched extracellular vesicles drive tumour metastasis in colorectal cancer via the RAC1/ABCA1 axis

**DOI:** 10.1002/ctm2.1591

**Published:** 2024-02-22

**Authors:** Weiwei Chen, Menghua Zhou, Bingjie Guan, Bowen Xie, Youdong Liu, Jiang He, Jingjing Zhao, Qian Zhao, Dongwang Yan

**Affiliations:** ^1^ Department of General Surgery Shanghai General Hospital Shanghai Jiao Tong University School of Medicine Shanghai China; ^2^ Department of General Surgery DongTai People's Hospital Dongtai Jiangsu China; ^3^ Department of Pathophysiology Key Laboratory of Cell Differentiation and Apoptosis of National Ministry of Education, Shanghai Jiao Tong University School of Medicine Shanghai China

**Keywords:** cholesterol metabolism, colorectal cancer, extracellular vesicles, metastasis, tumour‐associated macrophages

## Abstract

**Background:**

Metastasis accounts for the majority of deaths among patients with colorectal cancer (CRC). Here, the regulatory role of tumour‐associated macrophages (TAMs) in CRC metastasis was explored.

**Methods:**

Immunohistochemical (IHC) analysis of the TAM biomarker CD163 was conducted to evaluate TAM infiltration in CRC. Transwell assays and an ectopic liver metastasis model were established to evaluate the metastatic ability of tumour cells. RNA sequencing (RNA‐seq) and liquid chromatography‒mass spectrometry (LC‒MS) were applied to identify the differentially expressed genes and proteins in CRC cells and in TAM‐derived extracellular vesicles (EVs). Cholesterol content measurement, a membrane fluidity assay and filipin staining were performed to evaluate cholesterol efflux in CRC cells.

**Results:**

Our results showed that TAM infiltration is positively correlated with CRC metastasis. TAMs can facilitate the migration and invasion of MC‐38 and CT‐26 cells via EVs. According to the RNA‐seq data, TAM‐EVs increase cholesterol efflux and enhance membrane fluidity in CRC cells by regulating ABCA1 expression, thus affecting the motility of CRC cells. Mechanistically, DOCK7 packaged in TAM‐EVs can activate RAC1 in CRC cells and subsequently upregulate ABCA1 expression by phosphorylating AKT and FOXO1. Moreover, IHC analysis of ABCA1 in patients with liver‐metastatic CRC indicated that ABCA1 expression is significantly greater in metastatic liver nodules than in primary CRC tumours.

**Conclusions:**

Overall, our findings suggest that DOCK7 delivered via TAM‐EVs could regulate cholesterol metabolism in CRC cells and CRC cell metastasis through the RAC1/AKT/FOXO1/ABCA1 axis. DOCK7 could thus be a new therapeutic target for controlling CRC metastasis.

## Graphical abstract

Highlights

1. Tumour‐associated macrophages (TAMs) are abundant in colorectal cancer (CRC) and are correlated with metastasis.
2. TAM‐extracellular vesicles (EVs) could enhance CRC metastasis by transporting DOCK7.
3. DOCK7 packaged in TAM‐EVs could upregulate ABCA1 expression via RAC1 activation.
4. A novel interaction linking cholesterol metabolism to metastasis in CRC via TAM‐EVs was found.

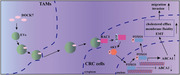

## 1 INTRODUCTION

Colorectal cancer (CRC), which ranks as the second leading cause in cancer‐related deaths, is the most prevalent malignant tumour in digestive system.^1^, ^2^ Metastasis is the primary obstacle to the successful treatment of CRC and mainly occurs in the liver. Liver metastasis is found in approximately 20% of CRC patients at diagnosis, and up to 50% of patients develop liver metastasis even after complete resection of the tumour in situ.^3^, ^4^ Current treatments, such as systemic chemotherapy, targeted therapies and immunotherapy, have shown limited therapeutic benefit in patients with metastatic CRC.^3^ Hence, there is an urgent need to identify the key mechanism and effective target underlying CRC metastasis.

In the CRC microenvironment, TAMs dominate the immune cell population, accounting for up to 50% of all immune cell population.^5^ There are generally two polarisations of TAMs. In contrast to M1‐TAMs, which play proinflammatory and antitumour roles, M2‐TAMs are closely related to anti‐inflammatory and protumour effects.^6^ Many studies have revealed that M2‐type macrophages are the most type of TAMs in the tumour microenvironment (TME).^7^ Accumulating research has revealed that TAMs can significantly modulate malignant phenotypes of tumours, such as proliferation,^8^ angiogenesis,^9^ motility^10^ and glycolysis.^11^ However, whether and how TAMs regulate metastasis in CRC have not been fully elucidated.

Communication between two or more types of cells in the TME is mediated mainly by soluble components, including secretory cytokines or extracellular vesicles (EVs). Previous studies of the regulatory effects of TAMs on tumour cells have focused mostly on secretory cytokines, such as chemokines and growth factors.^12^, ^13^ However, in recent years, attention to EVs has increased greatly, as EVs have a stable membrane structure containing a lipid bilayer loaded with proteins, nucleic acids, lipids and other cellular components.^14^ EVs can be secreted by all types of cells, which can be detected in all body fluids.^15^ Most previous studies related to EVs have focused on the regulation of tumour cell‐derived EVs, but little is known about TAM‐EVs in CRC metastasis.

In this study, we demonstrated that DOCK7 in TAM‐EVs could increase the migration and invasion abilities of MC‐38 and CT‐26 cells. Furthermore, mechanistic exploration revealed that DOCK7 packaged in TAM‐EVs activates RAC1 in CRC cells, and subsequently upregulates ABCA1 expression by phosphorylating AKT and FOXO1, ultimately regulating cholesterol metabolism and increasing membrane fluidity. We revealed that TAMs can reprogram cholesterol metabolism in CRC cells through the EV–DOCK7/RAC1/ABCA1 axis and lead to increased metastatic ability. Overall, our findings suggest that the DOCK7–ABCA1 axis is a new target for controlling metastasis of CRC.

## 2 MATERIALS AND METHODS

### 2.1 Patients and samples

CRC tissue microarray chips containing samples from 71 primary tumours were obtained from Shanghai General Hospital. In addition, 40 primary CRC tissues from CRC patients with liver metastasis were obtained at Shanghai General Hospital, Shanghai Jiao Tong University School of Medicine, China, between 2019 and 2022. All patients were diagnosed and underwent surgery at Shanghai General Hospital. Tissue specimens were used for detection of CD68, CD163 and ABCA1. The clinicopathological data of CRC patients were collected for analysis. All tissue specimens were collected from patients who provided informed consent.

### 2.2 Cell culture

The human embryonic kidney cell line HEK‐293T, murine colon cancer cell lines MC‐38 and CT‐26, murine fibroblast line L929 and murine leukaemic monocyte/macrophage line RAW 264.7 were obtained from the American Type Culture Collection (ATCC). Dulbecco's modified Eagle's medium (BasalMedia) supplemented with 10% fetal bovine serum (FBS; Sigma) and 1% penicillin/streptomycin (Invitrogen) were used to culture the above cells. The L929 and MC‐38 cell culture media were obtained by culturing the cells for 3 days and then collecting the culture supernatants.

The tibias and femurs of 6–8‐week‐old C57BL/6 mice were used to isolate the murine bone marrow‐derived macrophages (BMDMs). Briefly, mice were sacrificed, and intact tibias and femurs were obtained and rinsed to remove any muscle and soft tissue remaining on bones before being transferred to fresh culture medium. Furthermore, bone marrow cells were obtained by rinsing bones with a 1‐mL syringe and then seeding and culturing the obtained cells with complete medium containing mouse M‐CSF (30 ng/mL, PeproTech, 315‐02) or 30% L929‐CM for 6 days (M0 macrophages). Then, the BMDMs were cultured with 30% MC‐38‐CM for 3 days (TAMs). Then, the polarised BMDMs were collected for further experiments or cultured for extraction of EVs.

### 2.3 Isolation and identification of BMDM‐derived EVs

Before EV extraction, EV‐depleted FBS was obtained by centrifugation at 110 000 × *g* for 16 h at 4°C and filtering through a .22‐μm pore filter.^16^ After BMDMs were processed with MC‐38‐CM for 3 days, 10% EV‐depleted FBS was used for subsequent culture. The supernatant of the TAM and M0 macrophage cultures was collected every 3 days. The EVs in the supernatant were isolated by ultracentrifugation.^17^ Then, the resulting supernatant was ultracentrifuged at 110 000 × *g* for 90 min (Type 70 Ti, Beckman). The EVs precipitated through ultracentrifugation were subsequently resuspended in PBS for use. For verification, a morphological assessment strategy consisting of TEM (transmission electron microscopy) and NTA (nanoparticle tracking analysis) was used. The expression of EV markers was detected by Western blot analysis with antibodies such as anti‐CD9 (ABclonal, A1703), anti‐CD63 (ABclonal, A5271), anti‐ALIX (Proteintech, 12422‐1‐AP), anti‐TSG101 (Proteintech, 28283‐1‐AP) and anti‐HSP70 (ABclonal, A12948). A BCA assay was used to quantify the concentration of EVs. EVs were used at concentrations of 30 μg/mL for in vitro assays (Transwell, quantitation of cholesterol, filipin staining of cholesterol, membrane fluidity assay, immunofluorescence, Western blot analysis and qRT‒PCR analysis) and 50 μg/mL for in vivo assays (ectopic liver metastasis model). For immunogold labelling of EVs, isolated EVs were sent to Wuhan Misp Biotechnology Company for analysis. Immunogold staining of EVs was performed by incubating EVs with a mouse anti‐DOCK7 antibody (Proteintech, 13000‐1‐AP), followed by anti‐rabbit 10‐nm IgG gold (Sigma, G7402) as a secondary antibody.

### 2.4 Immunohistochemical staining

Immunohistochemical (IHC) staining was conducted following standard procedures, which included fixation, deparaffinisation, hydration, antigen retrieval, blocking and incubation with primary antibodies (4°C overnight) and biotinylated secondary antibodies. The experiment utilised the following primary antibodies: anti‐CD68 (Abcam, ab283654), anti‐ABCA1 (Abcam, ab18180) and anti‐CD163 (Abcam, ab182422). IHC staining was evaluated as previously reported.^9^

### 2.5 Labelling and tracking of BMDM‐derived EVs

PKH67 (Sigma, PKH67GL), a green fluorescent membrane dye, was used to label EVs purified from the culture media of TAMs and M0 macrophages (3 μL of PKH67 per 100 μg of EVs). The labelled EVs were extracted with ExoQuick Reagent (SBI, EXOTC50A‐1), resuspended in PBS and added to MC‐38 or CT‐26 cells for internalisation assays. A laser scanning confocal microscope (Nikon) was used to observe EV uptake after incubation for 6 h.

### 2.6 RNA interference

ShRNAs were obtained from SJTU‐SM (the scientific research platform) to generate a cell line with stable expression. Each constructed vector was cotransfected with the psPAX2 and pMD2.G into HEK293T cells (Lipofectamine 2000 transfection reagent, Invitrogen, 11668019) for 48 h to produce lentiviruses. Then, the lentivirus‐containing medium was filtered for further use. The shRNAs sequences are provided in Table S1.

### 2.7 Transwell assays

The 24‐well Transwell plates (Corning, 3422; pore size, 8 μm) were used for Transwell assays. For migration assays, educated or control MC‐38 cells (4 × 10^4^) or CT‐26 cells (2 × 10^4^) were plated in chambers (200 μL DMEM, upper), DMEM (600 μL, 10% FBS) was placed in the lower chambers for 48 h. For invasion assays, the membrane of each upper insert was precoated with Matrigel (50 μL, 1:10, Corning, 356234), and the incubation time was 24 h. Then, the cells were fixed and stained with 4% paraformaldehyde for 15 min and crystal violet (Beyotime, C0121) for 1 h. The cells were imaged in at least five random fields of view and counted.

### 2.8 Quantitation of cholesterol

The total cellular cholesterol concentration was quantified using a total cholesterol (TC) content assay kit (Solarbio, BC1985). Cells were suspended in isopropanol and lysed via ultrasonication (power 300 W, ultrasonication 2 s, interval 3 s, total time 3 min). Then, the lysed cells were centrifuged (10 000 × *g*, 4°C, 15 min). The working solution was used to mix with the resulting supernatant, and the samples were incubated in the dark (37°C, 15 min). The cholesterol concentration was determined by measuring the absorbance at 500 nm.

### 2.9 Filipin staining of cholesterol

Filipin III (Sigma, SAE0087) was diluted 1:20 with PBS and added to the cells for 2 h in the dark. After incubation, the staining was detected via laser scanning confocal microscopy (Nikon) using an excitation wavelength of 405 nm. All images were acquired with identical laser outputs, gains and offsets in each experiment.

### 2.10 Membrane fluidity assay

Cell membrane fluidity was evaluated according to the instruction (Abcam, ab189819, Membrane Fluidity Kit). The lipophilic pyrene probe was used to form excimers upon spatial interaction with the cell membrane. Membrane fluidity was assessed by calculating the ratio of monomer fluorescence (Em max, 370 nm) to excimer fluorescence (Em, 470 nm).

### 2.11 Immunofluorescence

For immunofluorescence assays, 24‐well plates were used to place MC‐38 and CT‐26 cells. After incubation with EVs for 48 h, the cells were fixed, washed and permeabilised. The target protein p‐FOXO1 was detected by incubation with primary antibodies (1:100 dilution; ABclonal, AP0172, 4°C, overnight). The next day, secondary antibodies (1:200 dilution; ABclonal, AS011) were applied for 1 h. After staining with DAPI, the coverslips were examined via laser scanning confocal microscopy (Nikon).

### 2.12 RAC1 activation pull‐down assay

The GTPase activity of RAC1 was detected by RAC1‐GTP pull‐down assays (Cytoskeleton, BK035). After treatment with TAM‐EVs (30 μg/mL), NSC23766 (50 μM) or ML‐097 (20 μM) cells were washed with PBS and collected in RIPA buffer. PAK‐PDB affinity beads (20 μg) were used to incubate equal protein extracts (200 μg). After incubation (1 h, 4°C), wash buffer was used to wash the beads gently for three times. Laemmli sample buffer (2×) was used to resuspend the beads conjugated to activated RAC1 proteins. Immunoblotting was performed to quantify active RAC1.

### 2.13 In vivo studies

To determine whether TAM‐EVs can facilitate the metastasis of CRC cells in vivo, a murine ectopic hepatic metastasis model was established through intrasplenic injection. In brief, 3 × 10^5^ CRC cells (100 μL PBS) treated with or without EVs (50 μg/mL) were inoculated into C57BL/6 and BALB/c mice via the spleens. For simvastatin treatment, the mice were treated every other day with simvastatin (30 mg/kg/day) by gavage. After tumours were allowed to form for 3 weeks, the livers were harvested and fixed for morphological analysis and HE staining.

### 2.14 Western blot analysis

RIPA buffer or SDS lysis buffer was used to lyse cells or EVs to extract proteins. The obtained protein extracts were mixed with loading buffer and separated on gels (6% or 10%). Then, the separated proteins were transferred onto .45 or .22 μm nitrocellulose membranes (Millipore, HATF04700) and blocked with 5% skim milk, and subsequently incubated with primary antibodies at 4°C overnight. Finally, the membranes were visualised with an enhanced chemiluminescence (ECL) detection kit (Vazyme, E423‐01) in a Digital Gel Image System 4200 (Tanon, 4200SF). The primary antibodies used here are given in Table S2.

### 2.15 RNA extraction and quantitative RT‒PCR analysis

VeZol Reagent (Vazyme, R411‐01) was used to extract total RNA from cells. AMV Reverse Transcriptase XL (Takara) was used to reverse total RNA transcribed into cDNA. Quantitative real‐time PCR was conducted by ChamQ SYBR qPCR Master Mix (Vazyme, Q311‐02) on an ABI 7300 PCR system (Applied Biosystems). The sequences of the primers used in our study are listed in Table S3.

### 2.16 RNA sequencing (RNA‐seq) and liquid chromatography‒mass spectrometry

The sequencing was conducted by Majorbio. Pooled RNA libraries were generated from the cells following the Illumina mRNA‐seq protocols with 1 μg of RNA from CT‐26 cells treated with PBS or TAM‐EVs. Differentially expressed genes (DEGs) between PBS‐ and TAM‐EV‐treated CT‐26 cells were identified. The RNA‐seq data have been deposited in the SRA database (PRJNA1026295).

The proteins in the EVs derived from M0 macrophages and TAMs were identified via mass spectrometry performed at Wayen Biotechnologies Company. The differentially expressed proteins in the analysis of liquid chromatography‒mass spectrometry (LC‒MS) are provided in Table S4.

### 2.17 Statistical analysis

All the statistical analyses were performed using GraphPad Prism 9, and the data are shown as the mean ± SD. Survival analysis was performed using the Kaplan–Meier method, and the data were compared using the log‐rank test. Unpaired two‐tailed Student's *t*‐test was applied for comparisons between two groups of normally distributed data. Comparisons among multiple groups were performed by two‐way ANOVA. In vitro experiments were performed at least twice or performed with independent samples. A *p*‐value of less than .05 was considered to indicate statistical significance.

## 3 RESULTS

### 3.1 TAMs in the primary CRC microenvironment are associated with metastasis

**FIGURE 1:**
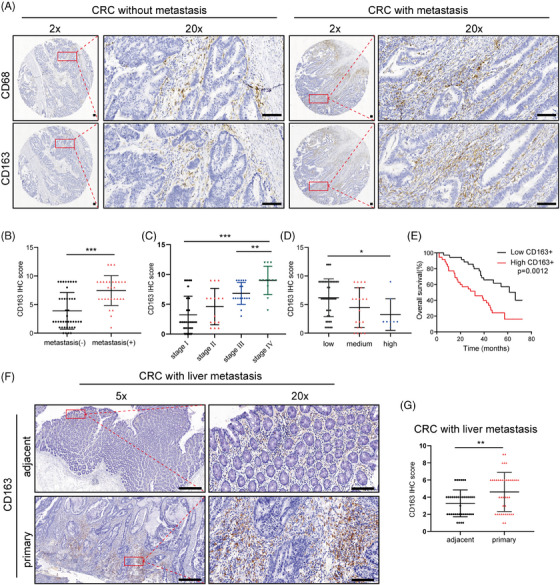
TAMs were associated with CRC metastasis. (A) Representative images of IHC staining for the TAM markers CD68 and CD163 in CRC patients with and without metastasis. The red boxes on the left are used to show higher magnifications of the area shown on the right. Scale bar: 100 μm. (B) The IHC score of CD163 in CRC patients with and without metastasis. (C) The expression of CD163 in CRC patients at different clinical stages. (D) The expression of CD163 in CRC patients with different degrees of tumour differentiation. (E) The overall survival probability of 71 CRC patients stratified according to CD163 expression. (F) Representative images of IHC staining for CD163 in CRC patients with liver metastasis. Scale bar: 100 μm. (G) The IHC score of CD163 in CRC patients with liver metastasis. The data are presented as the means ± SDs. *p < .05, **p < .01, ***p < .001.

To investigate the role of TAMs in CRC metastasis, 71 human CRC specimens and the corresponding clinicopathological data were collected. We selected the typical macrophage marker CD68 and the M2‐type TAM marker CD163 to evaluate the populations of macrophages and M2‐like TAMs by IHC analysis (Figure 1A). The results revealed that the recruitment of macrophages (CD68) and M2‐like TAMs (CD163) was greater in metastatic CRC tissues than in nonmetastatic tissues (Figure 1B and Figure S1A). In addition, the CD163 level, but not the CD68 level, was significantly higher in stage III and IV CRC tissues than in stage I and II CRC tissues (Figure 1C and Figure S1B). CRC patient tumour specimens with poorer differentiation also had elevated CD163 expression, but no significant difference was found in CD68 expression (Figure 1D and Figure S1C). By analysing the overall survival of the chosen CRC patients, we found that patients with high CD163 levels (IHC score 6−12) had poorer survival than patients with low CD163 levels (IHC score 0–4) (Figure 1E). Although there was no remarkable correlation between CD68 expression and overall survival, we found that patients in the CD68^low^CD163^low^ group had better survival than those in the CD68^high^CD163^high^ group (Figure S1D,E). According to the Kaplan‒Meier Plotter database,^18^ CRC patients with higher CD68 and CD163 expression levels had poorer overall survival and relapse‐free survival (Figure S1F–I). The most common site of CRC metastasis is the liver.^19^ To further investigate the correlation between TAMs and CRC liver metastasis, we verified the CD163 expression in 40 CRC patients diagnosed with liver metastasis. IHC analysis indicated that the expression level of CD163 was observed to be greater in primary CRC tissues (Figure 1F,G). Overall, these findings demonstrated that an abundant TAM population in the CRC microenvironment was correlated with metastasis and worse prognosis.

### 3.2 TAMs facilitate the migration and invasion of CRC cells through EVs

**FIGURE 2:**
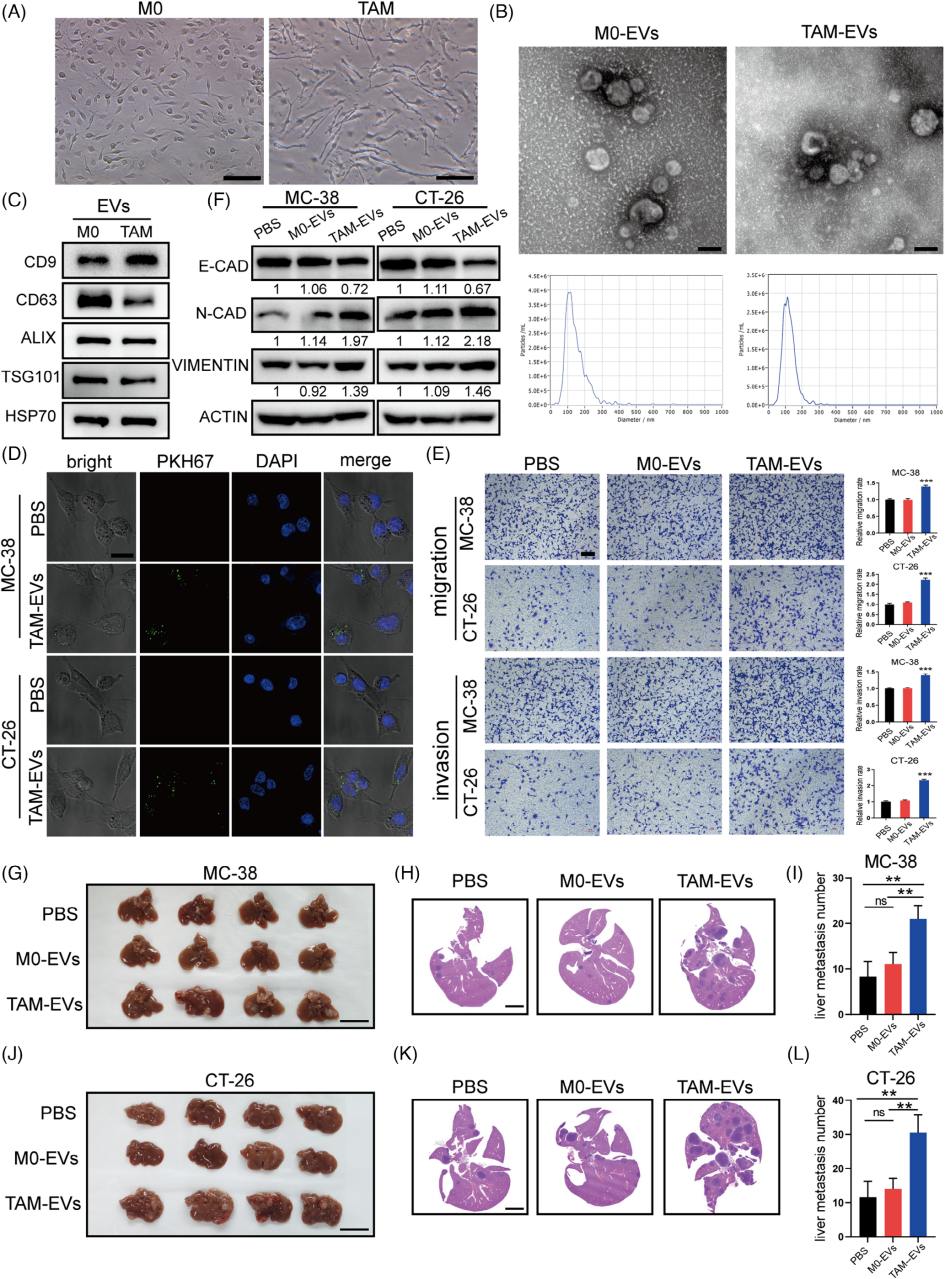
TAM‐EVs promote the migration and invasion of CRC cells in vitro and in vivo. (A) Typical morphological images of unpolarised M0 macrophages and MC‐38‐CM‐treated TAMs. Scale bar: 100 μm. (B) Representative structural images of M0‐EVs and TAM‐EVs acquired via transmission electron microscopy are shown. The concentration and size distributions of M0‐EVs and TAM‐EVs were determined via nanoparticle tracking analysis. Scale bar: 100 nm. (C) Western blot analysis of CD9, CD63, ALIX, TSG101 and HSP70 in M0‐EVs and TAM‐EVs. (D) Immunofluorescence images of PKH67‐labelled EVs (green) internalised by MC‐38 and CT‐26 cells. Scale bar: 20 μm. (E) Representative images of migration and invasion assays of M0‐ and TAM‐EV‐treated CRC cells and the quantification of migrated and invaded cells. Scale bar: 200 μm. (F) Western blot analysis of EMT markers in CRC cells treated with PBS, M0‐EVs or TAM‐EVs. (G–L) Representative morphological and HE staining images of livers from a murine ectopic hepatic metastasis model established through intrasplenic injection of PBS‐, M0‐EV‐ or TAM‐EV‐treated CRC cells. Scale bar: 2 cm. The quantification of liver metastatic nodules is shown on the right. The data are presented as the means ± SDs. *p < .05, **p < .01, ***p < .001.

To further investigate the crosstalk between TAMs and CRC cells, we first generated TAMs by culturing murine BMDMs processed with conditioned medium (CM) from MC‐38 murine colon cancer cells. The typical macrophage markers F4/80 and CD11b were evaluated via FACS to analyse the purity of the isolated macrophages (Figure S2A). By evaluating representative M1 markers (iNOS, CD86 and CD80) and M2 markers (CD206, Arg‐1 and IL‐10), we found that MC38‐CM‐educated TAMs tended to exhibit the M2 phenotype (Figure S2B–D). Morphologic images of uneducated macrophages (M0) and MC‐38‐CM‐educated TAMs showed that the TAMs displayed a more irregular and elongated shape than the M0 macrophages (Figure 2A). Next, we co‐cultured MC‐38 and CT‐26 murine CRC cells with collected M0‐CM and TAM‐CM. Via migration and invasion assays, we found that compared to M0‐CM, TAM‐CM significantly increased metastasis (Figure S2E). Since EVs can mediate intercellular communication by delivering their contents, we sought to explore whether EVs affect the TAM‐induced prometastatic impacts on CRC. First, EVs from M0 macrophages and TAMs were isolated by ultracentrifugation. TAM and NTA were performed to conduct a morphological assessment of the EVs from these two cell types. EVs from both cell types exhibited the expected cup‐shaped, lipid bilayer morphology and had typical diameters of 113.1 nm (98.4% of the M0‐EVs) and 119.6 nm (98.6% of the TAM‐EVs) (Figure 2B). The presence of EV marker proteins (CD9, CD63, ALIX, TSG101 and HSP70) were detected to further verify the identity of the isolated structures as EVs (Figure 2C). Next, we depleted EVs from TAM‐CM by ultracentrifugation and found that EV‐depleted TAM‐CM could not potentiate the migration and invasion of CRC cells as did untreated TAM‐CM (Figure S2E). Hence, here we demonstrated that TAMs could increase the metastatic ability of CRC cells by delivering EVs.

To verify whether M0‐EVs and TAM‐EVs can be taken up by CRC cells, the cells were incubated with PKH67‐labelled EVs. Confocal microscopy confirmed that both M0‐EVs and TAM‐EVs could be internalised by CRC cells (Figure 2D). Subsequently, we further demonstrated that TAM‐EVs significantly promoted the migration and invasion of CRC cells (Figure 2E). Moreover, we sought to explore whether epithelial–mesenchymal transition (EMT) is involved in this regulatory process. Western blot analysis was used to evaluate the expression of E‐cadherin, N‐cadherin and vimentin (EMT‐associated proteins). N‐cadherin and vimentin were upregulated and E‐cadherin was downregulated in CRC cells incubated with TAM‐EVs (Figure 2F). To gain further insight into whether TAM‐EVs could facilitate CRC metastasis in vivo, we generated a murine ectopic hepatic metastasis model established by intrasplenic injection of PBS‐, M0‐EV‐ or TAM‐EV‐treated MC‐38 cells into C57BL/6 mice and PBS‐, M0‐EV‐ or TAM‐EV‐treated CT‐26 cells into BALB/c mice. After tumours were allowed to form for 3 weeks, livers were harvested for morphological analysis and HE staining, and the results showed that the livers of mice injected with TAM‐EV‐treated cells had a larger metastatic burden than those of mice injected with PBS‐ or M0‐EV‐treated cells (Figure 2G–L). Taken together, the above results indicated that TAM‐EVs could promote CRC cell migration and invasion.

### 3.3 ABCA1 in CRC cells mediates the prometastatic effect of TAM‐EVs

**FIGURE 3:**
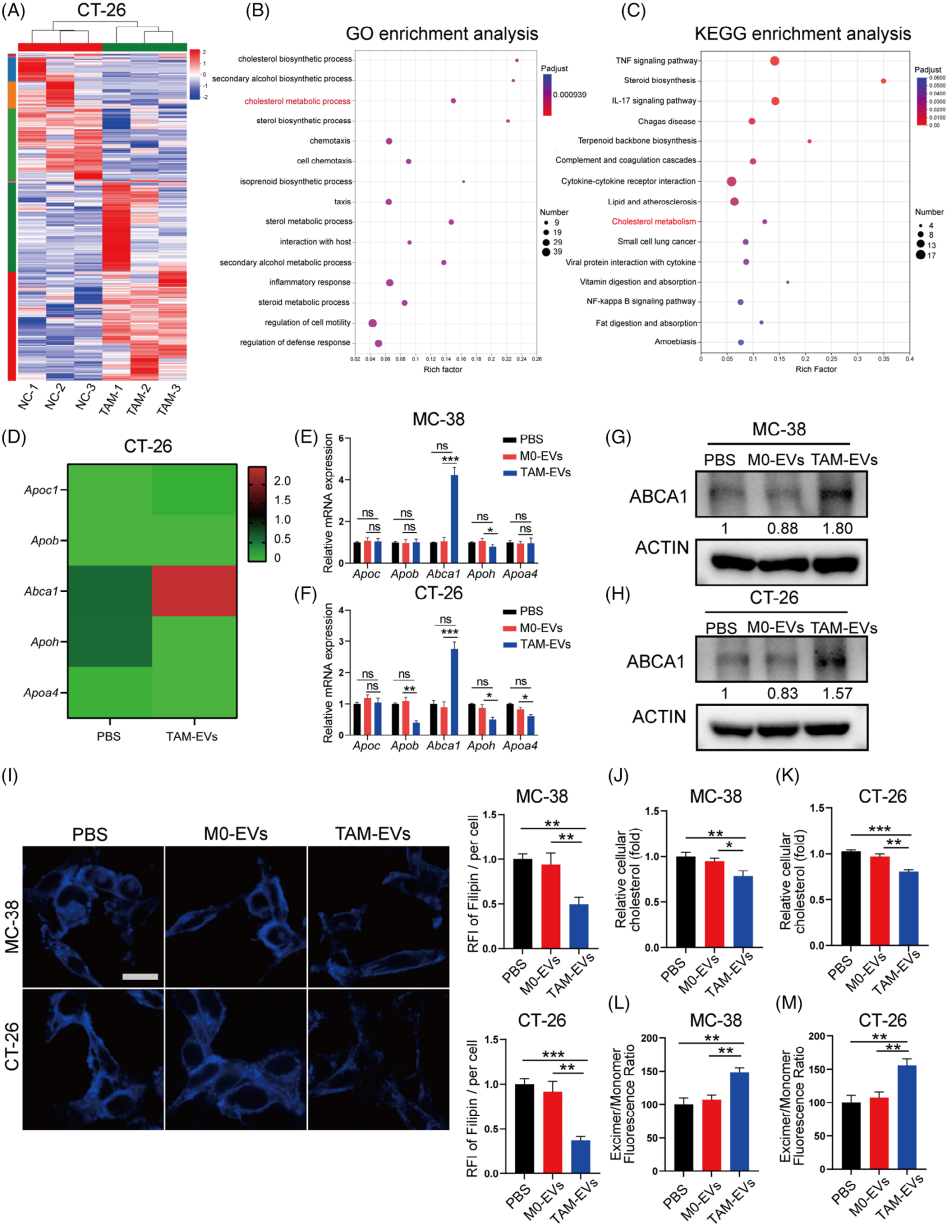
TAM‐EVs upregulate ABCA1 in CRC cells. (A) RNA‐seq analysis of CT‐26 cells treated with PBS or TAM‐EVs. (B and C) GO (B) and KEGG (C) enrichment analyses of 572 genes with significant differential expression between the TAM‐EV‐treated group and the control group, as determined by RNA‐seq analysis. (D) Genes (Apoc1, Apob, Abca1, Apoh and Apoa4) enriched in the cholesterol metabolism pathway according to the RNA‐seq data. (E–H) qRT‒PCR (E and F) and Western blot analyses (G and H) were applied to measure the mRNA and protein expression of ABCA1 in MC‐38 and CT‐26 cells. (I) Filipin staining was used to evaluate membrane cholesterol in CRC cells. Scale bar: 20 μm. The quantification of the mean fluorescence intensity (MFI) is shown on the right. (J and K) The total cholesterol content was measured in CRC cells treated with PBS or TAM‐EVs. (L and M) Membrane fluidity was evaluated in CRC cells treated with PBS or TAM‐EVs. The data are presented as the means ± SDs. *p < .05, **p < .01, ***p < .001.

To further elucidate the mechanism underlying the prometastatic effect of TAM‐EVs on CRC cells, RNA‐seq was performed on CT‐26 cells. Differential gene expression analysis revealed 324 upregulated genes and 248 downregulated genes (Figure 3A). GO and KEGG enrichment analyses of the above 530 DEGs were performed and indicated that TAM‐EVs are involved in multiple important pathways (Figure 3B,C). Among the enriched pathways, the cholesterol metabolism pathway attracted our attention because of its dysregulation, which is considered a hallmark of cancer. Cholesterol metabolism is reported to play a vital role in various aspects of tumour‐related processes such as the immune response, autophagy, chemoresistance and metastasis in many cancers, including CRC.^20^ However, the underlying regulatory mechanism of cholesterol metabolism remains largely unexplored, prompting us to further investigate whether cholesterol metabolic reprogramming mediated by TAM‐EVs could impact metastasis. Among the dysregulated genes involved in the cholesterol metabolism pathway as determined by our RNA‐seq analysis, ABCA1 was the most significantly upregulated (Figure 3D). We subsequently confirmed that treatment with TAM‐EVs was observed to upregulate ABCA1 expression in MC‐38 and CT‐26 cells (Figure 3E–H). Therefore, we hypothesised that TAM‐EVs exert their prometastatic effect on CRC cells through ABCA1. ABCA1 is a transporter of cellular phospholipids and cholesterol and participates in cancer metastasis in triple‐negative breast cancer.^21^ However, whether ABCA1 is responsible for metastasis of CRC remains unclear. Subsequently, we sought to further verify whether TAM‐EVs can regulate cholesterol metabolism in CRC cells. A filipin staining assay was performed to show that TAM‐EV‐treated MC‐38 and CT‐26 cells had lower membrane cholesterol contents (Figure 3I). The intracellular cholesterol content in CRC cells was also significantly decreased, as determined by a cellular cholesterol content assay (Figure 3J,K). Because membrane fluidity is strongly affected by cholesterol levels and is strongly related to metastasis, a membrane fluidity assay was also conducted in our cell models. After incubation with TAM‐EVs, MC‐38 and CT‐26 cells showed a higher excimer/monomer fluorescence ratio, indicating greater membrane fluidity (Figure 3L,M). In summary, TAM‐EVs could decrease both the membrane and intracellular cholesterol contents, possibly increasing the membrane fluidity of MC‐38 and CT‐26 cells.

**FIGURE 4:**
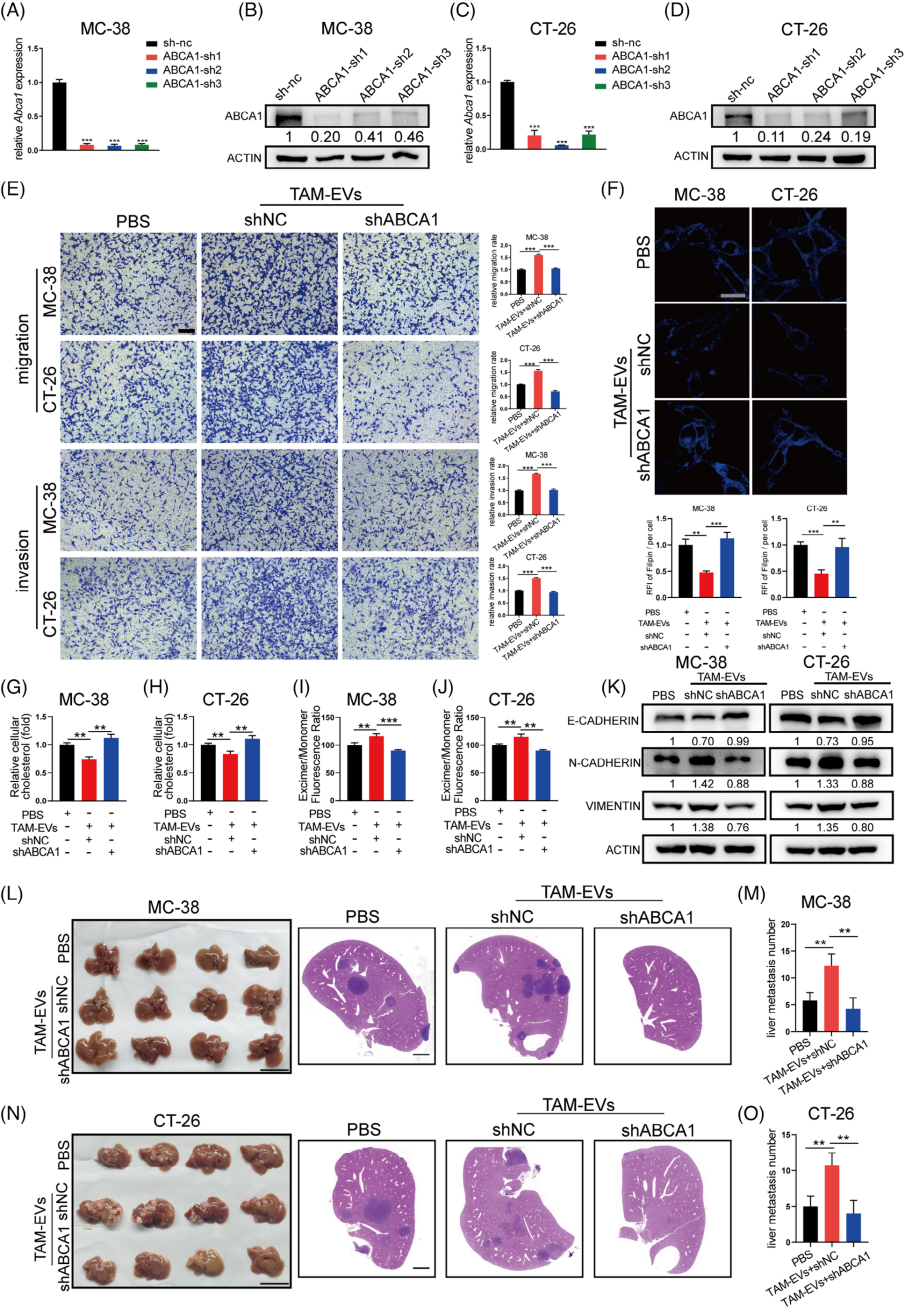
TAM‐EVs regulate cholesterol metabolism, membrane fluidity and metastatic ability via ABCA1 in CRC cells. (A–D) qRT‒PCR (A and C) and Western blot (B and D) analyses were conducted to determine the knockdown efficiency of the shRNA targeting ABCA1 in MC‐38 and CT‐26 cells. (E) Transwell assays were used to determine the effect of decreased ABCA1 expression on CRC cells; the quantification of the migrated and invaded cells is shown on the right. Scale bar: 200 μm. (F) Membrane cholesterol was evaluated by filipin staining in CRC cells. Scale bar: 20 μm. The quantification of the mean fluorescence intensity (MFI) is shown on the right. (G and H) The total cholesterol content was measured in CRC cells treated with TAM‐EVs and transduced with or without shABCA1. (I and J) Membrane fluidity was evaluated in control and shABCA1‐transfected CRC cells treated with TAM‐EVs. (L–O) Representative morphological and HE staining images of livers from a murine ectopic hepatic metastasis model established by intrasplenic injection of CRC cells transfected with empty vector or the shABCA1 plasmid and subsequent treatment with PBS or TAM‐EVs. The quantification of metastatic nodules in the liver is shown on the right. Scale bar: 2 cm. The data are presented as the means ± SDs. *p < .05, **p < .01, ***p < .001.

To further confirm whether ABCA1 in CRC cells can mediate the regulatory effects of TAM‐EVs on metastasis and cholesterol metabolism, we first designed three shRNA fragments to knockdown ABCA1 in MC‐38 and CT‐26 cells. The results showed that all three fragments markedly decreased ABCA1 expression, and we selected the fragment with the highest knockdown efficiency (sh‐2) for subsequent experiments (Figure 4A–D). Transwell assays were performed to show that shABCA1 treatment attenuated the promotive effects of TAM‐EVs on the metastatic ability of MC‐38 and CT‐26 cells (Figure 4E). Filipin staining and cellular cholesterol content assays revealed that both the membrane and intracellular cholesterol contents were increased in the TAM‐EV/shABCA1 group (Figure 4F–H). TAM‐EV/shABCA1 MC‐38 and CT‐26 cells also exhibited decreased membrane fluidity (Figure 4I,J). These results indicated that TAM‐EVs could regulate cholesterol metabolism by altering ABCA1 expression. In addition, the TAM‐EV/shABCA1 group showed a trend towards a mitigated EMT phenotype (Figure 4K). We further established an ectopic hepatic metastasis model to verify the TAM‐EV‐ABCA1 regulatory relationship in vivo. Morphological analysis and HE staining revealed that TAM‐EV‐/shABCA1‐treated MC‐38 and CT‐26 cells formed fewer metastatic nodules in the liver (Figure 4L–O). In summary, by regulating ABCA1, TAM‐EVs could increase cell migration and invasion through cholesterol efflux and increased membrane fluidity.

### 3.4 TAMs activate RAC1 in CRC cells to regulate metastatic ability and cholesterol metabolism via transfer of DOCK7

**FIGURE 5:**
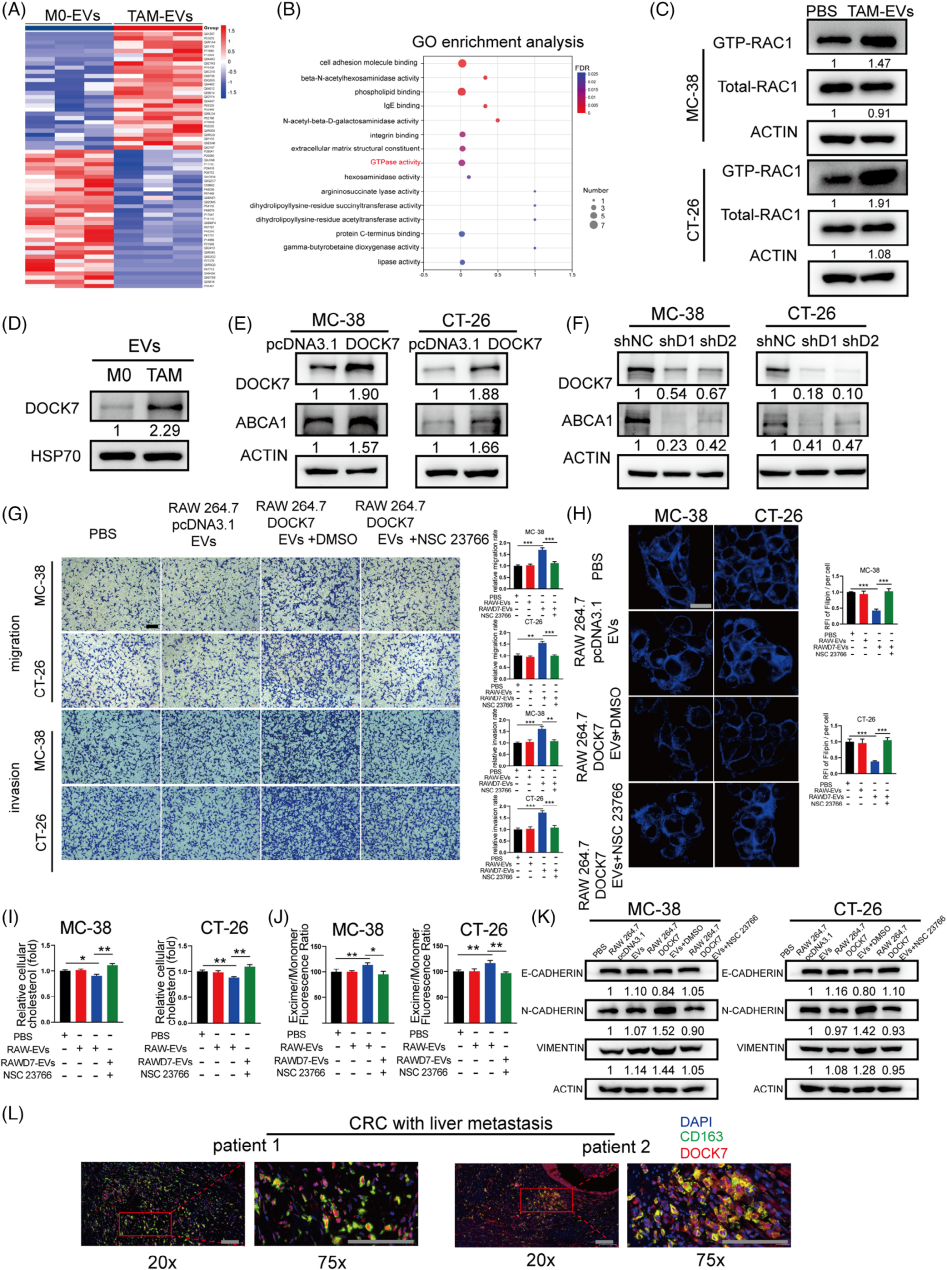
DOCK7 packaged in TAM‐EVs regulates cholesterol metabolism and the metastatic phenotype via activation of RAC1 in CRC cells. (A) Heatmap showing the differential expression profile of TAM‐EVs compared to M0‐EVs, as determined via LC‒MS analysis. (B) GO enrichment analysis of differentially abundant proteins between TAM‐EVs and M0‐EVs. (C) A GTP‐RAC1 pull‐down assay was applied to detect the activation of RAC1 in CRC cells after PBS or TAM‐EV treatment. (D) Western blot analysis was used to evaluate DOCK7 expression in M0‐EVs and TAM‐EVs. (E and F) The overexpression (E) and knockdown (F) efficiency of DOCK7 and subsequent ABCA1 expression were verified by Western blot analysis. (G–K) Migration and invasion assays (G), filipin staining (H), total cholesterol content measurement (I), a membrane fluidity assay (J) and Western blot analysis of EMT markers (K) were conducted to evaluate CRC cells treated with or without RAW264.7‐NC/OEDOCK7‐EVs combined with NSC23766. (L) Double immunofluorescence staining for the TAM markers CD163 and DOCK7 in CRC patients with liver metastasis. Scale bar: 100 μm. The data are presented as the means ± SDs. *p < .05, **p < .01, ***p < .001.

To identify the potential components of TAM‐EVs that promote metastasis and regulate cholesterol metabolism in CRC cells, LC‒MS was performed to distinguish differentially abundant proteins between M0‐EVs and TAM‐EVs (Figure 5A). GO enrichment analysis showed that the GTPase activity, cell adhesion molecule binding and integrin binding pathways were the main pathways enriched in the differentially abundant proteins (Figure 5B). Here, the GTPase activity pathway attracted our attention because RAC1, a classical small GTPase, was reported to upregulate ABCA1 expression, although the underlying mechanism was not fully elucidated.^22^ The RAC1 GST pull‐down assay demonstrated that incubating MC‐38 and CT‐26 cells with TAM‐EVs led to an increased GTP‐RAC1 level (Figure 5C). Therefore, we hypothesised that RAC1 in CRC cells could mediate the regulatory effect of TAM‐EVs. To verify this hypothesis, the RAC1 inhibitor NSC23766 and RAC activator ML‐097 were used to regulate the activity of RAC1. A subsequent RAC1 GST pull‐down assay showed that NSC23766 decreased the GTP‐RAC1 level and that ML‐097 significantly activated RAC (Figure S3A,B). Moreover, ABCA1 expression was shown to be regulated by RAC1 activity (Figure S3A,B). Subsequently, migration and invasion assays revealed that the decrease in GTP‐RAC1 mitigated the prometastatic effect of TAM‐EVs (Figure S3C). Filipin staining and cellular cholesterol content assays showed that both the intracellular and membrane cholesterol contents were markedly increased in cells treated with TAM‐EVs in combination with NSC23766 (Figure S3D–F). Moreover, a lower degree of membrane fluidity was observed in the TAM‐EV/NSC23766 group than in the TAM‐EV/DMSO group (Figure S3G,H). These results demonstrated that TAM‐EVs could exert their regulatory effects on the oncogenic phenotype by activating RAC1 in CRC cells.

Among the proteins with significantly increased abundances in TAM‐EVs according to our LC‒MS data, DOCK7 was notable for its high expression level and its ability to activate RAC1 as a guanine nucleotide exchange factor (GEF). In addition, high expression level of DOCK7 was found in ovarian cancer and tended to be a potential target for chemotherapy.^23^ However, the role of DOCK7 in CRC remains largely unknown. First, we confirmed that DOCK7 was enriched in TAM‐EVs compared with M0‐EVs (Figure 5D). We subsequently sought to determine whether DOCK7 can modulate ABCA1 expression in CRC cells. PcDNA3.1‐DOCK7 and a shRNA against DOCK7 were used to overexpress and silence DOCK7, respectively. Western blot analyses verified the DOCK7 overexpression and knockdown efficiencies. We verified that DOCK7 was responsible for GEF activity, but had no regulatory effect on the total RAC1 protein level (Figure S3I,J). Interestingly, the expression level of ABCA1 was correlated with the alteration in DOCK7 expression positively, which indicated that ABCA1 might be regulated intracellularly by DOCK7 (Figure 5E,F). We proceeded to validate the correlations among DOCK7, RAC1 and ABCA1 expression in clinical samples. We observed a notable positive correlation between DOCK7 and ABCA1 expression in CRC patients from GEPIA database (Figure S4A). However, no significant correlation was found between DOCK7 and RAC1 expression or between RAC1 and ABCA1 expression (Figure S4B,C). These findings further suggested that the upregulation of ABCA1 by DOCK7 is not achieved through modulation of RAC1 expression but rather through modulation of RAC1 activation via its GEF activity.

Furthermore, we sought to explore whether DOCK7 in EVs can regulate cholesterol efflux and the metastatic ability of CRC cells by altering RAC1 activity. As BMDMs were too difficult to transfect, we used a mouse leukemic monocyte/macrophage line, RAW264.7 cells, for subsequent EV delivery studies. Similar to the results described above, MC‐38‐CM increased DOCK7 expression in RAW264.7‐EVs (Figure S5B). In addition, pcDNA3.1‐DOCK7 and shRNA against DOCK7 were transfected into RAW264.7 cells, and DOCK7 expression in cells and their derived EVs was verified by Western blot analysis (Figure S5A,B). We subsequently found that EVs derived from DOCK7‐overexpressing RAW264.7cells (oeDOCK7‐EVs) facilitated the metastatic ability of CRC cells via Transwell assays, which were alleviated when the cells were cotreated with NSC23766 (Figure 5G). The intracellular and membrane cholesterol contents were also decreased after cotreatment with oeDOCK7‐EVs, and NSC23766 treatment reversed this decrease (Figure 5H,I). In addition, NSC23766 treatment attenuated the decrease in membrane fluidity in CRC cells, which was increased by oeDOCK7‐EVs (Figure 5J). Western blot analysis also revealed that NSC23766 treatment suppressed the EMT phenotype, which was induced by oeDOCK7‐EVs (Figure 5K). Moreover, compared with MC‐38 CM‐treated RAW264.7/shNC‐EVs, CRC cells treated with MC‐38 CM‐treated RAW264.7/shDOCK7‐EVs exhibited decreased migration and invasion abilities, increased intracellular and membrane cholesterol contents, decreased membrane fluidity and an attenuated EMT phenotype. In vivo assays also indicated that CRC cells treated with CM‐treated RAW264.7/shDOCK7‐EVs formed significantly fewer metastatic nodules in the liver. However, the above changes in CRC cells were reversed by combined treatment with ML‐097 (Figure S5C–O). In summary, the above results indicated that DOCK7 packaged in EVs could regulate cholesterol metabolism and the metastatic ability by activating RAC1 in CRC cells.

As RAC1 is reported to be recognised and activated by binding to DOCK7,^24^ we subsequently sought to explore whether EV–DOCK7 exhibits the same binding activity in CRC cells and if so, to determine the subcellular location of this interaction. By immunogold labelling electron microscopy, we observed that DOCK7 anchored to the TAM‐EV surface (Figure S6A). Subsequently, the mCherry‐DOCK7 plasmid was transfected into RAW264.7 cells, mCherry‐DOCK7‐EVs were collected, and immunofluorescence assays were performed. EV–DOCK7 was found to be internalised by CRC cells and colocalised with intracellular RAC1 (Figure S6B), suggesting that DOCK7 packaged in EVs could bind to intracellular RAC1 for further activation. Because the above experiments were carried out in mouse cells, we next sought to explore whether TAM and DOCK7 are colocalised in clinical human CRC specimens. Immunofluorescence staining for CD163 and DOCK7 showed that CD163 was obviously colocalised with DOCK7 in CRC patients with liver metastasis (Figure 5L).

In summary, these findings demonstrated that DOCK7 transferred via TAM‐EVs could bind to intracellular RAC1 to regulate cellular metastatic ability and cholesterol metabolism.

### 3.5 DOCK7 packaged in TAM‐EVs mediates ABCA1 expression through the AKT‐FOXO1 signalling pathway

**FIGURE 6:**
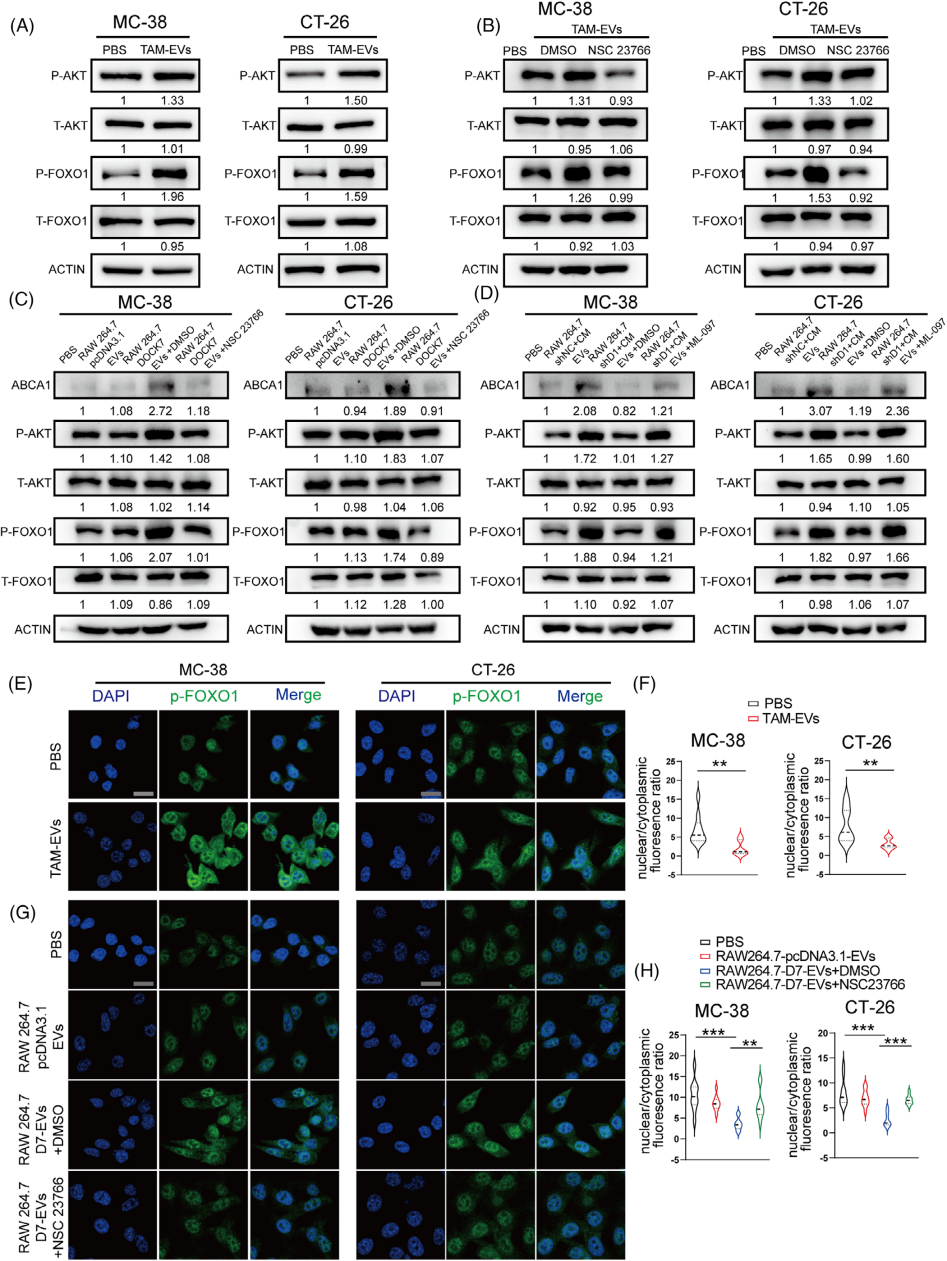
DOCK7 delivered via TAM‐EVs could upregulate ABCA1 expression via the RAC1/AKT/FOXO1 axis in CRC cells. (A and B) AKT/FOXO1 signalling pathway proteins were analysed by Western blotting in CRC cells treated with or without TAM‐EVs (A) and NSC23766 (B). (C and D) Western blotting was used to analyse ABCA1 and AKT/FOXO1 pathway proteins in CRC cells treated with or without RAW264.7‐NC/OEDOCK7‐EVs combined with NSC23766 (C) or RAW264.7‐shNC/shDOCK7+MC38‐CM combined with ML‐097 (D). (E–H) Immunofluorescence staining was conducted to evaluate the localisation and staining intensity of p‐FOXO1 in CRC cells treated with or without TAM‐EVs (E and F) or treated with or without RAW264.7‐NC/OEDOCK7‐EVs combined with NSC23766 (G and H). Scale bar: 20 μm. The data are presented as the means ± SDs. *p < .05, **p < .01, ***p < .001.

As our above findings demonstrated that TAM‐EV‐derived DOCK7 could activate RAC1 to upregulate the expression of ABCA1, we further sought to explore the specific mechanism underlying this regulation. A variety of factors can mediate the transcriptional regulation of ABCA1. Among these factors, the AKT/FOXO1 axis has been reported to significantly regulate ABCA1 expression. Phosphorylated AKT can phosphorylate FOXO1, after which p‐FOXO1 can be translocated, thereby relieving the transcriptional inhibition of ABCA1 by FOXO1.^25^ In addition, RAC1 has been found to phosphorylate AKT in various cancers.^26^, ^27^ Therefore, we hypothesised that TAM‐EV‐derived DOCK7‐RAC1 signalling could regulate ABCA1 expression via AKT/FOXO1. First, Western blot analysis showed that MC‐38 and CT‐26 cells treated with TAM‐EVs had significantly increased p‐AKT and p‐FOXO1 levels (Figure 6A). We subsequently found that NSC23766 treatment markedly mitigated the ability of TAM‐EVs to increase p‐AKT and p‐FOXO1 expression levels in CRC cells (Figure 6B). Similarly, the RAW264.7 cells were transfected with pcDNA3.1‐DOCK7 and shDOCK7 plasmids. Treatment with oeDOCK7‐RAW264.7‐EVs increased the protein levels of ABCA1, p‐AKT and p‐FOXO1 in CRC cells, and these increases were inhibited by combined treatment with NSC23766 (Figure 6C). In addition, compared with control‐EV‐treated cells, cells incubated with MC‐38 CM‐treated RAW264.7/shDOCK7‐EVs exhibited lower protein levels of ABCA1, p‐AKT and p‐FOXO1. However, these decreases were reversed by treatment with the RAC1 agonist ML‐097 in CRC cells (Figure 6D). We further conducted an immunofluorescence assay to verify the nuclear‐cytoplasmic translocation of p‐FOXO1. The results showed that TAM‐EVs markedly increased the content and staining intensity of p‐FOXO1 in the cytoplasm (Figure 6E,F). Moreover, NSC23766 alleviated the increase in cytoplasmic p‐FOXO1 induced by oeDOCK7‐RAW264.7‐EV treatment in CRC cells (Figure 6G,H). In summary, the TAM‐EV–DOCK7/RAC1 axis could mediate the upregulation of ABCA1 via the AKT/FOXO1 signalling pathway.

### 3.6 ABCA1 is highly expressed in metastatic CRC and has potential utility as a new therapeutic target

**FIGURE 7:**
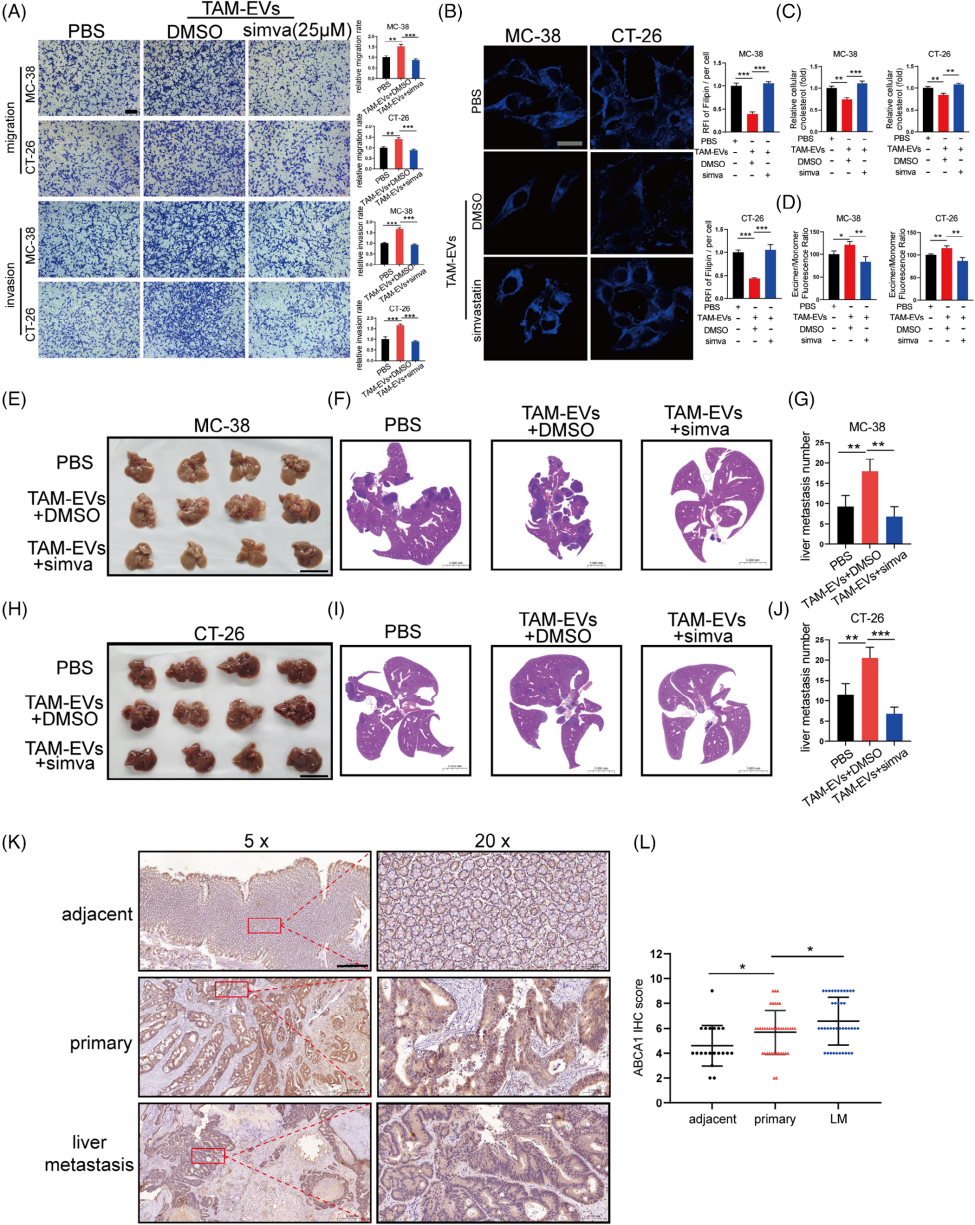
Simvastatin attenuated the prometastatic effect of TAM‐EVs via inhibition of ABCA1. (A) Representative images of migration and invasion assays of CRC cells treated with TAM‐EVs with or without simvastatin, accompanied by the quantification of migrated and invaded cells. Scale bar: 200 μm. (B) Filipin staining was used to evaluate membrane cholesterol in CRC cells treated with TAM‐EVs with or without simvastatin. Scale bar: 20 μm. The quantification of the mean fluorescence intensity (MFI) is shown on the right. (C) The total cholesterol content was measured in CRC cells treated with TAM‐EVs with or without simvastatin. (D) Membrane fluidity was evaluated in CRC cells treated with TAM‐EVs with or without simvastatin. (E–J) Representative morphological (E and H) and HE staining images (F and I) of livers from a murine ectopic hepatic metastasis model established by intrasplenic injection of CRC cells treated with or without TAM‐EVs followed by intragastric administration of simvastatin every other day. The quantification of liver metastatic nodules is shown on the right (G and J). Scale bar: 5 cm. (K and L) IHC analysis of ABCA1 expression in adjacent normal tissues, primary CRC tissues and liver metastatic specimens from CRC patients with liver metastasis. Scale bar: 500 μm (K). The quantification of the IHC score for ABCA1 is shown on the right (L). The data are presented as the means ± SDs. *p < .05, **p < .01, ***p < .001.

To further verify whether ABCA1 can be used as a therapeutic target to control CRC metastasis, simvastatin, an inhibitor of ABCA1, was selected for use in the following experiments. Simvastatin was also reported to inhibit tumour metastasis in various cancers.^28^, ^29^ First, we incubated MC‐38 and CT‐26 cells with three concentrations (10, 25 and 50 μM) of simvastatin. The results revealed that a concentration of 25 μM markedly inhibited ABCA1 mRNA and protein expression, while 50 μM did not have a stronger inhibitory effect (Figure S7A,B). Therefore, we chose 25 μM for subsequent experiments. Migration and invasion assays showed that simvastatin treatment significantly attenuated the prometastatic ability of TAM‐EVs (Figure 7A). In addition, MC‐38 and CT‐26 cells incubated with simvastatin had higher intracellular and membrane cholesterol contents and lower membrane fluidity than did those treated with TAM‐EVs (Figure 7B–D). An ectopic hepatic metastasis model was established to verify whether simvastatin can exert its inhibitory effect in vivo. After the spleens of C57BL/6 mice and BALB/c mice were injected with TAM‐EV‐treated MC‐38 and CT‐26 cells, we administered simvastatin every other day by gavage. After 3 weeks, mice were sacrificed and livers were harvested for morphological analysis and HE staining. The results indicated that mice treated with simvastatin had significantly fewer liver metastatic nodules (Figure 7E–J). Subsequently, 40 CRC patients who had liver metastasis and underwent surgery were enrolled in our study, and we collected primary tumour and matched liver metastasis specimens and adjacent normal tissues. The results showed that the ABCA1 expression level in the liver metastatic nodules was significantly higher than that in the adjacent normal tissues and primary tumours by IHC staining for ABCA1 (Figure 7K,L). In addition, higher expression level of ABCA1 was negatively correlated with better relapse‐free survival and overall survival according to the Kaplan‒Meier Plotter database (Figure S8A,B). Overall, the above results demonstrated that ABCA1 is abundant in metastatic CRC and is a promising new therapeutic target.

## 4 DISCUSSION

Despite the increase in the prognosis on account of improved diagnosis and treatment, metastatic CRC remains a lethal disease with an approximately 14% of 5‐year survival rate.^30^ Identification of the complex mechanism underlying CRC metastasis is crucial for determining strategies to precisely target metastatic CRC. The invasion‐metastasis cascade has been described in CRC.^31^ The first and primary step of this cascade in CRC is the detachment of CRC cells from adjacent normal cells through changes in the EMT‐associated proteins.^32^ Multiple factors can regulate metastasis, including intracellular factors (cell heterogeneity, gene mutation and EMT) and extracellular factors (the TME). Due to its rich cellular composition and highly developed cellular communication network, the role of the TME in tumour metastasis has received increasing attention. TAMs have been reported to regulate various cancer‐related processes. For instance, TAMs can inhibit antitumour immunity through regulatory T‐cell (T‐reg) activation by IL‐10 and TGF‐β secretion.^33^ Recent research has shown that the infiltration of CD206 TAMs into the tumour core could promote CRC tumour growth by shaping an inhibitory TME.^34^ However, the specific role of TAMs in CRC metastasis has not been fully elucidated. EVs participate in the intercellular communication between cells in the TME by virtue of their complete bilayer membrane structure and their ability for stable long‐distance transport of their contents.^35^ Most related previous research concentrated on the regulation of TAM polarisation and depolarisation by EVs secreted by tumour cells, but there is limited knowledge regarding the effect of TAM‐EVs on tumour cells.^36^, ^37^ In our study, we first demonstrated that the expression of CD163 (a classical marker of TAMs) was strongly related to CRC metastasis and that TAM‐EVs could facilitate the metastatic ability of CRC cells. Although EVs are found to be loaded with a variety of components, such as RNA, proteins and metabolites, most current studies have focused on noncoding RNAs.^9^ However, whether TAM‐EVs have a regulatory role in the metastasis of CRC through their encapsulated proteins remains unclear.

Through LC‒MS, we found that DOCK7 was more abundant in TAM‐EVs than in M0‐EVs. DOCK7 is involved in the dedicator of cytokinesis (DOCK) family. The DOCK family consists of 11 members that contain two DOCK homology regions (DHRs), and DOCK7 belongs to the Dock‐C subfamily.^38^, ^39^ As classical GEFs, the members of the DOCK family can markedly activate Rho GTPase (RhoA, RAC1, CDC42, etc.) signalling by facilitating GDP/GTP exchange.^24^ To date, few studies have investigated the biological functions of DOCK7. However, previous research has shown its critical role in regulating axonal growth in the nervous system. DOCK7 participates in axon development through inhibitory phosphorylation of the microtubule destabilising protein Stathmin/Op18 on S16 via Rac activation.^40^ In addition, DOCK7, regulated by SET, could activate Rac1 to promote the migration of ESCC cells and facilitate metastasis in mice.^41^ DOCK7 has also been reported to be highly expressed in ovarian cancer, and is recruited into the nucleus by MDC1 to participate in the DNA damage response through the RAC1/CDC42/PAK1 module. Deletion of DOCK7 can increase the sensitivity of ovarian cancer cells to chemotherapy.^23^ However, whether DOCK7 exerts crucial effects on CRC cells remains unclear. Our results herein indicated that DOCK7 packaged in TAM‐EVs could activate RAC1 in CRC cells, and subsequently upregulate ABCA1 expression by phosphorylating AKT and FOXO1.

Our study is the first to show that TAM‐EVs can significantly increase the ABCA1 expression in CRC cells. Subsequently, mechanistic experiments demonstrated that DOCK7 in TAM‐EVs could regulate ABCA1 via the RAC1/AKT/FOXO1 axis. ABCA1 is a widely expressed transmembrane protein that is involved in cholesterol homeostasis. The most commonly reported function of ABCA1 is the transport of intracellular free cholesterol and phospholipids outside the cell membrane, where they combine with apolipoproteins.^42^ Cholesterol metabolism in tumour cells is very complex and involves a dynamic process of change. Cell membrane integrity is highly regulated by cellular cholesterol homeostasis, which can modulate the activity of membrane‐anchored signalling pathways. In addition, rapidly proliferating tumour cells are reported to have increased intracellular cholesterol levels.^43^ Previous studies have shown that ABCA1 plays dual roles across tumour types. In breast cancer and prostate cancer, decreased ABCA1 expression facilitates cancer cell proliferation.^44^, ^45^ On the other hand, ABCA1 is reportedly a diagnostic marker because of its high expression in TNBC tissues.^46^ In addition, high ABCA1 expression in ovarian cancer is related to poor prognosis and promotes malignant cell phenotypes.^47^ Cholesterol metabolism also plays an important role in CRC metastasis, but whether ABCA1 regulates this process has not been determined. In our study, we found that TAM‐EVs promoted cholesterol efflux and increased membrane fluidity via the modulation of ABCA1 expression. The increase in membrane fluidity controlled by membrane cholesterol is also closely related to EMT and increases in cancer cell motility and invasiveness. Moreover, the IHC assays of tissues from patients with liver‐metastatic CRC indicated that the expression level of ABCA1 was markedly greater in metastatic nodules. However, whether cholesterol released from CRC cells can promote CRC metastasis or other malignant phenotypes via cooperation with other factors in the TME needs further study.

The EV–DOCK7/ABCA1 regulatory axis links cholesterol metabolism and tumour metastasis in CRC via TAM‐EVs. Knowledge regarding this intercellular communication‐mediated metabolic reprogramming is crucial for further therapeutic targets to control CRC metastasis. By investigating the effects of TAM‐EVs on CRC cells, we considered multiple possible therapeutic approaches to control CRC metastasis, as follows: (i) targeted treatment for DOCK7‐high TAMs. Although this is an attractive strategy, as little is known about whether DOCK7 is enriched on the surface of the TAM membrane, it is necessary to develop more accurate localisation probes or EV secretion inhibitors to target TAMs with high DOCK7 expression. (ii) Treatment strategies that interfere with EV production and release by DOCK7‐high TAMs and with EV internalisation by CRC cells. Before this strategy can be implemented, further experiments are needed to confirm whether DOCK7 localises to the membrane of EVs. (iii) Treatments targeting the RAC1/AKT/FOXO1/ABCA1 regulatory axis in CRC cells to maintain the expression of ABCA1 at a stable level. (iv) Metabolic intervention with ABCA1‐mediated cholesterol efflux, aiming to reverse the EMT phenotype of CRC cells caused by increased membrane fluidity, thereby inhibiting the motility and invasion of CRC cells. All the above‐mentioned treatment strategies might be used in combination with existing chemotherapies in the hope of achieving a better therapeutic effect.

In our research, there are still some unknowns that need further resolution. First, the mechanism of DOCK7 upregulation in TAMs and the mechanism through which DOCK7 is packaged into EVs remain unclear and need further study. Second, DOCK7 is certainly not the only functional molecule in TAM‐EVs that can regulate tumour metastasis. We believe that DOCK7 can promote metastasis and metabolic reprogramming together with other molecules; thus, other influential EV components need to be further explored and verified. In addition, the understanding of whether TAM‐EVs contribute to other pathological processes of CRC, such as oxidative phosphorylation and the formation of the premetastatic niche, is still very limited, and further exploration is required.

## 5 CONCLUSIONS

**FIGURE 8:**
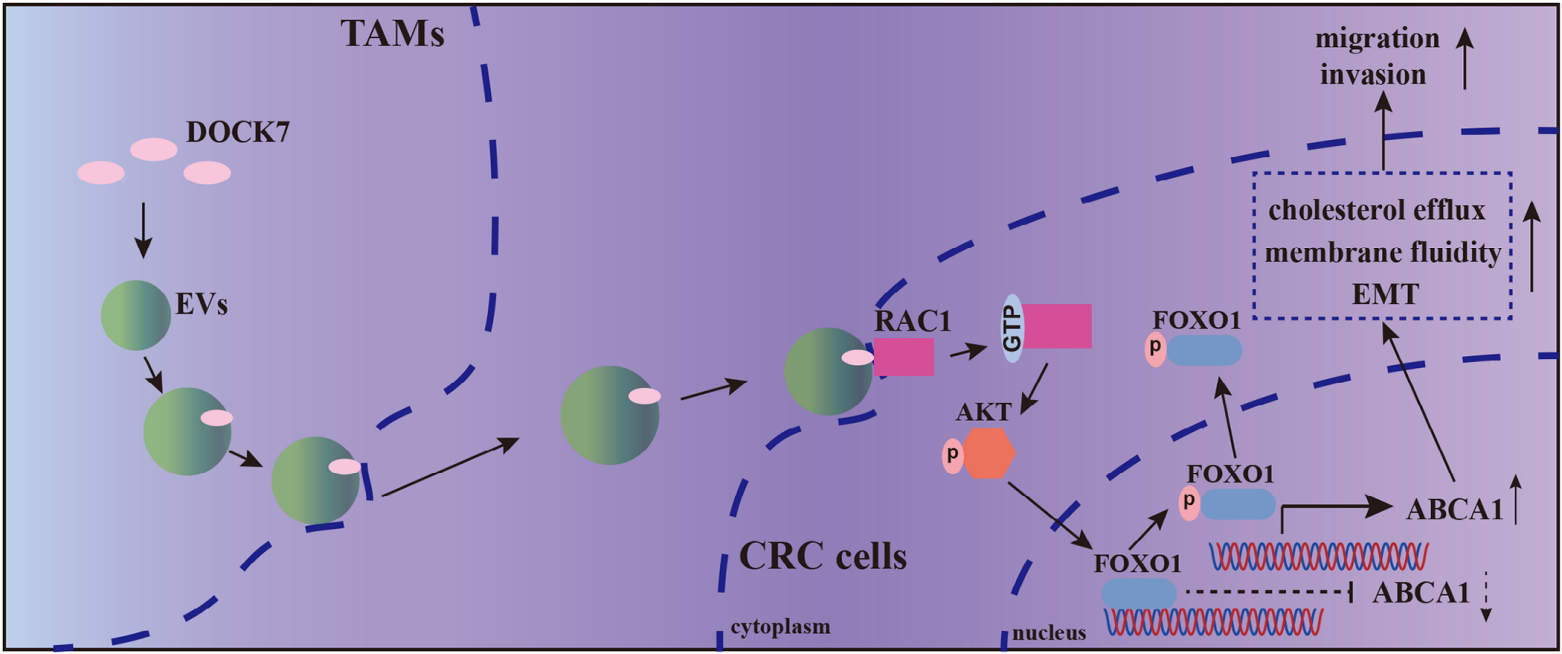
DOCK7 in EVs mediates intercellular communication between TAMs and CRC cells. The schematic diagram shows that DOCK7 packaged in TAM‐EVs could regulate cholesterol metabolism and the metastatic ability of CRC cells by mediating ABCA1 expression via the RAC1/AKT/FOXO1 pathway.

In conclusion, we found that TAMs are abundant in the CRC microenvironment and are correlated with metastasis. EVs derived from TAMs could increase the migration and invasion by transferring DOCK7 to CRC cells. Further mechanistic exploration indicated that DOCK7 packaged in TAM‐EVs could activate RAC1 in CRC cells, and subsequently upregulate ABCA1 expression by phosphorylating AKT and FOXO1, ultimately regulating cholesterol metabolism and increasing membrane fluidity to regulate the motility and invasiveness of CRC cells (Figure 8). Our findings reveal a novel interaction linking cholesterol metabolism to metastasis in CRC via TAM‐EVs, which might be expected to become a new therapeutic target to control CRC metastasis.

## AUTHOR CONTRIBUTIONS

Weiwei Chen: conceptualisation, data curation, formal analysis, investigation, methodology, project administration and writing—original draft. Menghua Zhou: data curation, formal analysis, investigation, methodology and validation. Bingjie Guan: data curation, formal analysis, investigation, software and validation. Bowen Xie: investigation, software and validation. Youdong Liu: data curation and validation. Jiang He: software and resources. Jingjing Zhao: investigation, software and resources. Qian Zhao: conceptualisation, formal analysis, resources and supervision. Dongwang Yan: conceptualisation, funding acquisition, methodology, formal analysis, resources and supervision.

## CONFLICT OF INTEREST STATEMENT

The authors declare they have no potential conflicts of interest.

## ETHICS STATEMENT

This study was approved by the Ethics Committee of Shanghai General Hospital, Shanghai Jiao Tong University School of Medicine (2020SQ150), and performed in accordance with all applicable guidelines and regulations. The animal procedures were conducted according to the standards of the National Institutes of Health Guide for the Care and Use of Laboratory Animals and approved by Laboratory Animal Ethics Committee (2022AW015).

## Supporting information


**Figure S1** Clinical significance of CD68 and CD163 in CRC patients. (A–D) Relationships between CD68 expression and metastasis, stage, differentiation status and survival in CRC patients. (E) Survival analysis of CD68^low^CD163^low^ patients and CD68^high^CD163^high^ patients. (F–I) The expression levels of CD68 (F and G) and CD163 (H and I) were positively correlated with poor overall survival and relapse‐free survival according to the Kaplan‒Meier Plotter database.

**Figure S2** TAMs promote the migration and invasion of CRC cells through Evs. (A–C) FACS analysis of the murine macrophage markers F4/80 and CD11b (A) and the TAM marker CD206 (B). The mean fluorescence intensity of CD206 is shown on the right (C). (D) qRT‒PCR was used to measure the levels of M2 markers (CD206, Arg‐1 and IL‐10) and M1 markers (INOS, CD86 and CD80). (E) Representative images of migration and invasion assays of CRC cells treated with EV‐depleted CM or untreated CM, accompanied by the quantification of migrated and invaded cells on the right. Scale bar: 200 μm. The data are presented as the means ± SDs. *p < .05, **p < .01, ***p < .001.

**Figure S3** TAM‐EVs regulate metastatic ability, cholesterol metabolism and membrane fluidity in CRC cells via activation of RAC1. (A and B) RAC1 activation and ABCA1 expression were evaluated by Western blot analysis in MC‐38 and CT‐26 cells treated with TAM‐EVs combined with NSC23766 or ML‐097. (C) Transwell assays were used to determine the effects of NSC23766 on the migration and invasion of CRC cells treated with TAM‐EVs, and the quantification of migrated and invaded cells is shown on the right. Scale bar: 200 μm. (D) Membrane cholesterol was evaluated by filipin staining in CRC cells treated with TAM‐EVs with or without NSC23766. Scale bar: 20 μm. The quantification of the mean fluorescence intensity (MFI) is shown at the bottom. (E and F) The total cholesterol content was measured in CRC cells treated with TAM‐EVs with or without NSC23766. (G and H) Membrane fluidity was evaluated in CRC cells treated with TAM‐EVs with or without NSC23766. (I and J) A GTP‐RAC1 pull‐down assay was performed to detect RAC1 activation in MC‐38 and CT‐26 cells with overexpression or silencing of DOCK7. The data are presented as the means ± SDs. *p < .05, **p < .01, ***p < .001.

**Figure S4** Correlations among DOCK7, RAC1 and ABCA1 expression in CRC clinical samples. (A) A positive correlation was observed between DOCK7 and ABCA1 expression in CRC patients based on the GEPIA database. (B and C) No significant correlation was found between DOCK7 and RAC1 expression or between RAC1 and ABCA1 expression in CRC patients.

**Figure S5** DOCK7 in RAW264.7‐EVs could regulate the protumour phenotype mediated by ABCA1 via activation of RAC1. (A and B) Western blot analysis was used to evaluate the expression of DOCK7 in RAW264.7 cells transfected with pcDNA3.1‐DOCK7 or transduced with shRNA against DOCK7 (A) and in the corresponding RAW264.7‐EVs (B). (C–I) Migration and invasion assays (C), filipin staining (D), total cholesterol content measurement (E and F), a membrane fluidity assay (G and H) and Western blot analysis of EMT markers (I) were conducted to evaluate CRC cells treated with or without MC‐38 CM‐treated RAW264.7‐shNC/shDOCK7‐EVs alone or combined with ML‐097. Representative morphological (J and K) and HE staining images (L and M) of livers from a murine ectopic hepatic metastasis model established by intrasplenic injection of CRC cells treated with or without MC‐38 CM‐treated RAW264.7‐shNC/shDOCK7‐EVs. The quantification of liver metastatic nodules is shown at the bottom (N and O). Scale bar: 1 cm. The data are presented as the means ± SDs. *p < .05, **p < .01, ***p < .001.

**Figure S6** DOCK7 packaged in EVs could bind to intracellular RAC1 in CRC cells. (A) Immunogold labelling electron microscopy showing the location of the DOCK7 protein in TAM‐EVs. Scale bar: 100 nm. (B) EV‐packaged DOCK7 was found to bind to intracellular RAC1 in CRC cells. Scale bar: 10 μm.

**Figure S7** Simvastatin decreased the mRNA and protein levels of ABCA1 in CRC cells. (A and B) qRT‒PCR and Western blot analyses were conducted to measure the mRNA and protein expression levels of ABCA1 in MC‐38 (A) and CT‐26 (B) cells treated with simvastatin. The data are presented as the means ± SDs. *p < .05, **p < .01, ***p < .001.

**Figure S8** ABCA1 expression is positively correlated with poor prognosis in CRC patients. (A and B) The expression level of ABCA1 was positively correlated with poor overall survival (A) and relapse‐free survival (B) according to the Kaplan‒Meier Plotter database.

**Supporting Table S1** cDNA target sequences of the shRNAs.
**Supporting Table S2** Primary antibodies used for Western blot analysis.
**Supporting Table S3** Primer sequences used for real‐time PCR.

Supporting Information

## Data Availability

The RNA‐seq data have been deposited in the SRA database (PRJNA1026295). The corresponding author can provide the data and materials used in the current study upon reasonable request.

## References

[1] Siegel RL , Miller KD , Fuchs HE , Jemal A . Cancer statistics, 2022. CA Cancer J Clin. 2022;72(1):7‐33. doi:10.3322/caac.21708 35020204

[2] Sung H , Ferlay J , Siegel RL , et al. Global cancer statistics 2020: GLOBOCAN estimates of incidence and mortality worldwide for 36 cancers in 185 countries. CA Cancer J Clin. 2021;71(3):209‐249. doi:10.3322/caac.21660 33538338

[3] Biller LH , Schrag D . Diagnosis and treatment of metastatic colorectal cancer: a review. JAMA. 2021;325(7):669‐685. doi:10.1001/jama.2021.0106 33591350

[4] Dekker E , Tanis PJ , Vleugels JLA , Kasi PM , Wallace MB . Colorectal cancer. Lancet. 2019;394(10207):1467‐1480. doi:10.1016/s0140-6736(19)32319-0 31631858

[5] Zhong X , Chen B , Yang Z . The role of tumor‐associated macrophages in colorectal carcinoma progression. Cell Physiol Biochem. 2018;45(1):356‐365. doi:10.1159/000486816 29402795

[6] Allavena P , Sica A , Solinas G , Porta C , Mantovani A . The inflammatory micro‐environment in tumor progression: the role of tumor‐associated macrophages. Crit Rev Oncol Hematol. 2008;66(1):1‐9. doi:10.1016/j.critrevonc.2007.07.004 17913510

[7] Solinas G , Germano G , Mantovani A , Allavena P . Tumor‐associated macrophages (TAM) as major players of the cancer‐related inflammation. J Leukocyte Biol. 2009;86(5):1065‐1073. doi:10.1189/jlb.0609385 19741157

[8] Li X , Tang M . Exosomes released from M2 macrophages transfer miR‐221‐3p contributed to EOC progression through targeting CDKN1B. Cancer Med. 2020;9(16):5976‐5988. doi:10.1002/cam4.3252 32590883 PMC7433826

[9] Liu Q , Zhao E , Geng B , et al. Tumor‐associated macrophage‐derived exosomes transmitting miR‐193a‐5p promote the progression of renal cell carcinoma via TIMP2‐dependent vasculogenic mimicry. Cell Death Dis. 2022;13(4):382. doi:10.1038/s41419-022-04814-9 35443741 PMC9021253

[10] Yang C , Dou R , Wei C , et al. Tumor‐derived exosomal microRNA‐106b‐5p activates EMT‐cancer cell and M2‐subtype TAM interaction to facilitate CRC metastasis. Mol Ther. 2021;29(6):2088‐2107. doi:10.1016/j.ymthe.2021.02.006 33571679 PMC8178444

[11] Chen F , Chen J , Yang L , et al. Extracellular vesicle‐packaged HIF‐1α‐stabilizing lncRNA from tumour‐associated macrophages regulates aerobic glycolysis of breast cancer cells. Nat Cell Biol. 2019;21(4):498‐510. doi:10.1038/s41556-019-0299-0 30936474

[12] Chen J , Yao Y , Gong C , et al. CCL18 from tumor‐associated macrophages promotes breast cancer metastasis via PITPNM3. Cancer Cell. 2011;19(4):541‐555. doi:10.1016/j.ccr.2011.02.006 21481794 PMC3107500

[13] Condeelis J , Pollard JW . Macrophages: obligate partners for tumor cell migration, invasion, and metastasis. Cell. 2006;124(2):263‐266. doi:10.1016/j.cell.2006.01.007 16439202

[14] Liu Y , Gu Y , Cao X . The exosomes in tumor immunity. Oncoimmunology. 2015;4(9):e1027472. doi:10.1080/2162402x.2015.1027472 26405598 PMC4570093

[15] Bastos N , Ruivo CF , da Silva S , Melo SA . Exosomes in cancer: use them or target them? Semin Cell Dev Biol. 2018;78:13‐21. doi:10.1016/j.semcdb.2017.08.009 28803894

[16] Théry C , Amigorena S , Raposo G , Clayton A . Isolation and characterization of exosomes from cell culture supernatants and biological fluids. Curr Protoc Cell Biol. 2006. Chapter 3:Unit 3.22. doi:10.1002/0471143030.cb0322s30 18228490

[17] Zhang H , Freitas D , Kim HS , et al. Identification of distinct nanoparticles and subsets of extracellular vesicles by asymmetric flow field‐flow fractionation. Nat Cell Biol. 2018;20(3):332‐343. doi:10.1038/s41556-018-0040-4 29459780 PMC5931706

[18] Győrffy B . Discovery and ranking of the most robust prognostic biomarkers in serous ovarian cancer. GeroScience. 2023;45(3):1889‐1898. doi:10.1007/s11357-023-00742-4 36856946 PMC10400493

[19] Tsilimigras DI , Brodt P , Clavien PA , et al. Liver metastases. Nat Rev Dis Primers. 2021;7(1):27. doi:10.1038/s41572-021-00261-6 33859205

[20] Xiao M , Xu J , Wang W , et al. Functional significance of cholesterol metabolism in cancer: from threat to treatment. Exp Mol Med. 2023;55(9):1982‐1995. doi:10.1038/s12276-023-01079-w 37653037 PMC10545798

[21] Wang Y , Zhou X , Lei Y , et al. NNMT contributes to high metastasis of triple negative breast cancer by enhancing PP2A/MEK/ERK/c‐Jun/ABCA1 pathway mediated membrane fluidity. Cancer Lett. 2022;547:215884. doi:10.1016/j.canlet.2022.215884 35988817

[22] Fond AM , Lee CS , Schulman IG , Kiss RS , Ravichandran KS . Apoptotic cells trigger a membrane‐initiated pathway to increase ABCA1. J Clin Invest. 2015;125(7):2748‐2758. doi:10.1172/jci80300 26075824 PMC4563683

[23] Gao M , Guo G , Huang J , et al. DOCK7 protects against replication stress by promoting RPA stability on chromatin. Nucleic Acids Res. 2021;49(6):3322‐3337. doi:10.1093/nar/gkab134 33704464 PMC8034614

[24] Kukimoto‐Niino M , Ihara K , Murayama K , Shirouzu M . Structural insights into the small GTPase specificity of the DOCK guanine nucleotide exchange factors. Curr Opin Struct Biol. 2021;71:249‐258. doi:10.1016/j.sbi.2021.08.001 34507037

[25] Lyu J , Imachi H , Iwama H , Zhang H , Murao K . Insulin‐like growth factor 1 regulates the expression of ATP‐binding cassette transporter A1 in pancreatic beta cells. Horm Metab Res. 2016;48(5):338‐344. doi:10.1055/s-0035-1569272 26743528

[26] Huang WK , Chen Y , Su H , et al. ARHGAP25 inhibits pancreatic adenocarcinoma growth by suppressing glycolysis via AKT/mTOR pathway. Int J Biol Sci. 2021;17(7):1808‐1820. doi:10.7150/ijbs.55919 33994864 PMC8120455

[27] Liang J , Liu Q , Xia L , et al. Rac1 promotes the reprogramming of glucose metabolism and the growth of colon cancer cells through upregulating SOX9. Cancer Sci. 2023;114(3):822‐836. doi:10.1111/cas.15652 36369902 PMC9986058

[28] Yin W , Zhao Y , Kang X , et al. BBB‐penetrating codelivery liposomes treat brain metastasis of non‐small cell lung cancer with EGFR(T790M) mutation. Theranostics. 2020;10(14):6122‐6135. doi:10.7150/thno.42234 32483443 PMC7255027

[29] Li Q , Wang M , Hu Y , et al. MYBL2 disrupts the Hippo‐YAP pathway and confers castration resistance and metastatic potential in prostate cancer. Theranostics. 2021;11(12):5794‐5812. doi:10.7150/thno.56604 33897882 PMC8058714

[30] Rumpold H , Niedersüß‐Beke D , Heiler C , et al. Prediction of mortality in metastatic colorectal cancer in a real‐life population: a multicenter explorative analysis. BMC Cancer. 2020;20(1):1149. doi:10.1186/s12885-020-07656-w 33238958 PMC7691098

[31] Shin AE , Giancotti FG , Rustgi AK . Metastatic colorectal cancer: mechanisms and emerging therapeutics. Trends Pharmacol Sci. 2023;44(4):222‐236. doi:10.1016/j.tips.2023.01.003 36828759 PMC10365888

[32] Mendonsa AM , Na TY , Gumbiner BM . E‐cadherin in contact inhibition and cancer. Oncogene. 2018;37(35):4769‐4780. doi:10.1038/s41388-018-0304-2 29780167 PMC6119098

[33] Mantovani A , Sozzani S , Locati M , Allavena P , Sica A . Macrophage polarization: tumor‐associated macrophages as a paradigm for polarized M2 mononuclear phagocytes. Trends Immunol. 2002;23(11):549‐555. doi:10.1016/s1471-4906(02)02302-5 12401408

[34] Yin Y , Liu B , Cao Y , et al. Colorectal cancer‐derived small extracellular vesicles promote tumor immune evasion by upregulating PD‐L1 expression in tumor‐associated macrophages. Adv Sci. 2022;9(9):2102620. doi:10.1002/advs.202102620 PMC8948581 35356153

[35] Thakur A , Parra DC , Motallebnejad P , Brocchi M , Chen HJ . Exosomes: small vesicles with big roles in cancer, vaccine development, and therapeutics. Bioact Mater. 2022;10:281‐294. doi:10.1016/j.bioactmat.2021.08.029 34901546 PMC8636666

[36] Wang F , Niu Y , Chen K , et al. Extracellular vesicle‐packaged circATP2B4 mediates M2 macrophage polarization via miR‐532‐3p/SREBF1 axis to promote epithelial ovarian cancer metastasis. Cancer Immunol Res. 2023;11(2):199‐216. doi:10.1158/2326-6066.Cir-22-0410 36512324 PMC9896028

[37] Ono K , Sogawa C , Kawai H , et al. Triple knockdown of CDC37, HSP90‐alpha and HSP90‐beta diminishes extracellular vesicles‐driven malignancy events and macrophage M2 polarization in oral cancer. J Extracell Vesicles. 2020;9(1):1769373. doi:10.1080/20013078.2020.1769373 33144925 PMC7580842

[38] Côté JF , Vuori K . GEF what? Dock180 and related proteins help Rac to polarize cells in new ways. Trends Cell Biol. 2007;17(8):383‐393. doi:10.1016/j.tcb.2007.05.001 17765544 PMC2887429

[39] Gadea G , Blangy A . Dock‐family exchange factors in cell migration and disease. Eur J Cell Biol. 2014;93(10‐12):466‐477. doi:10.1016/j.ejcb.2014.06.003 25022758

[40] Watabe‐Uchida M , John KA , Janas JA , Newey SE , Van Aelst L . The Rac activator DOCK7 regulates neuronal polarity through local phosphorylation of stathmin/Op18. Neuron. 2006;51(6):727‐739. doi:10.1016/j.neuron.2006.07.020 16982419

[41] Yuan X , Wang X , Gu B , et al. Directional migration in esophageal squamous cell carcinoma (ESCC) is epigenetically regulated by SET nuclear oncogene, a member of the inhibitor of histone acetyltransferase complex. Neoplasia. 2017;19(11):868‐884. doi:10.1016/j.neo.2017.08.003 28938158 PMC5608591

[42] Jacobo‐Albavera L , Domínguez‐Pérez M , Medina‐Leyte DJ , González‐Garrido A , Villarreal‐Molina T . The role of the ATP‐binding cassette A1 (ABCA1) in human disease. Int J Mol Sci. 2021;22(4):1593. doi:10.3390/ijms22041593 33562440 PMC7915494

[43] Kolanjiappan K , Ramachandran CR , Manoharan S . Biochemical changes in tumor tissues of oral cancer patients. Clin Biochem. 2003;36(1):61‐65. doi:10.1016/s0009-9120(02)00421-6 12554062

[44] Fukuchi J , Hiipakka RA , Kokontis JM , et al. Androgenic suppression of ATP‐binding cassette transporter A1 expression in LNCaP human prostate cancer cells. Cancer Res. 2004;64(21):7682‐7685. doi:10.1158/0008-5472.Can-04-2647 15520169

[45] Schimanski S , Wild PJ , Treeck O , et al. Expression of the lipid transporters ABCA3 and ABCA1 is diminished in human breast cancer tissue. Horm Metab Res. 2010;42(2):102‐109. doi:10.1055/s-0029-1241859 19902402

[46] Pan H , Zheng Y , Pan Q , et al. Expression of LXR‑β, ABCA1 and ABCG1 in human triple‑negative breast cancer tissues. Oncol Rep. 2019;42(5):1869‐1877. doi:10.3892/or.2019.7279 31432185 PMC6775801

[47] Hedditch EL , Gao B , Russell AJ , et al. ABCA transporter gene expression and poor outcome in epithelial ovarian cancer. J Natl Cancer Inst. 2014;106(7):dju149. doi:10.1093/jnci/dju149 24957074 PMC4110473

