# Supplementary figures and images for: Tumour‐associated macrophage‐derived DOCK7‐enriched extracellular vesicles drive tumour metastasis in colorectal cancer via the RAC1/ABCA1 axis
Source: Clin Transl Med. 2024 Feb 22;14(2):e1591. doi: 10.1002/ctm2.1591 (PMC10883245; doi:10.1002/ctm2.1591)

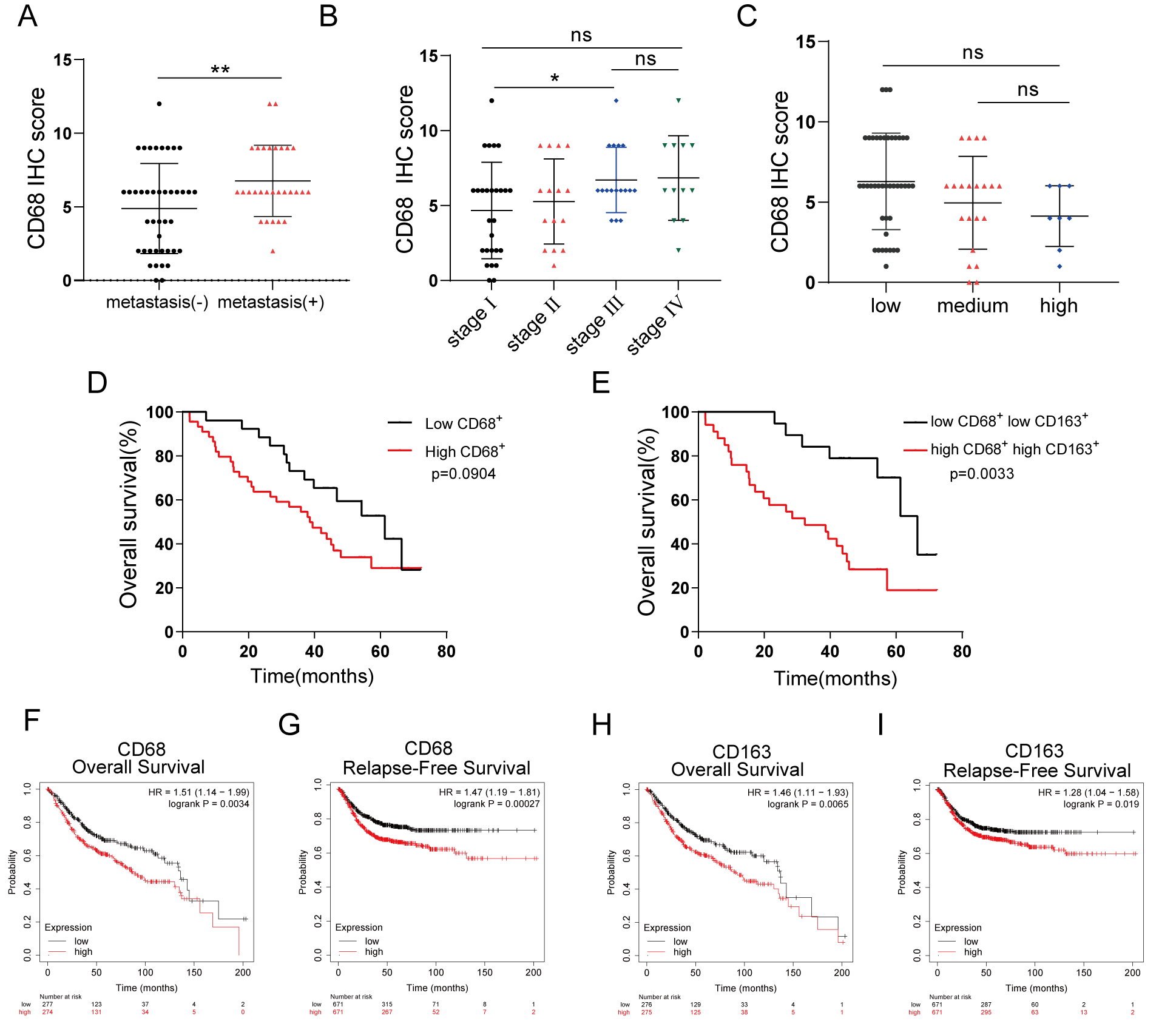

Supplement: Supplementary file 1 — Figure S1 Clinical significance of CD68 and CD163 in CRC patients. (A–D) Relationships between CD68 expression and metastasis, stage, differentiation status and survival in CRC patients. (E) Survival analysis of CD68lowCD163low patients and CD68highCD163high patients. (F–I) The expression levels of CD68 (F and G) and CD163 (H and I) were positively correlated with poor overall survival and relapse‐free survival according to the Kaplan‒Meier Plotter database. [file CTM2-14-e1591-s009.tif]

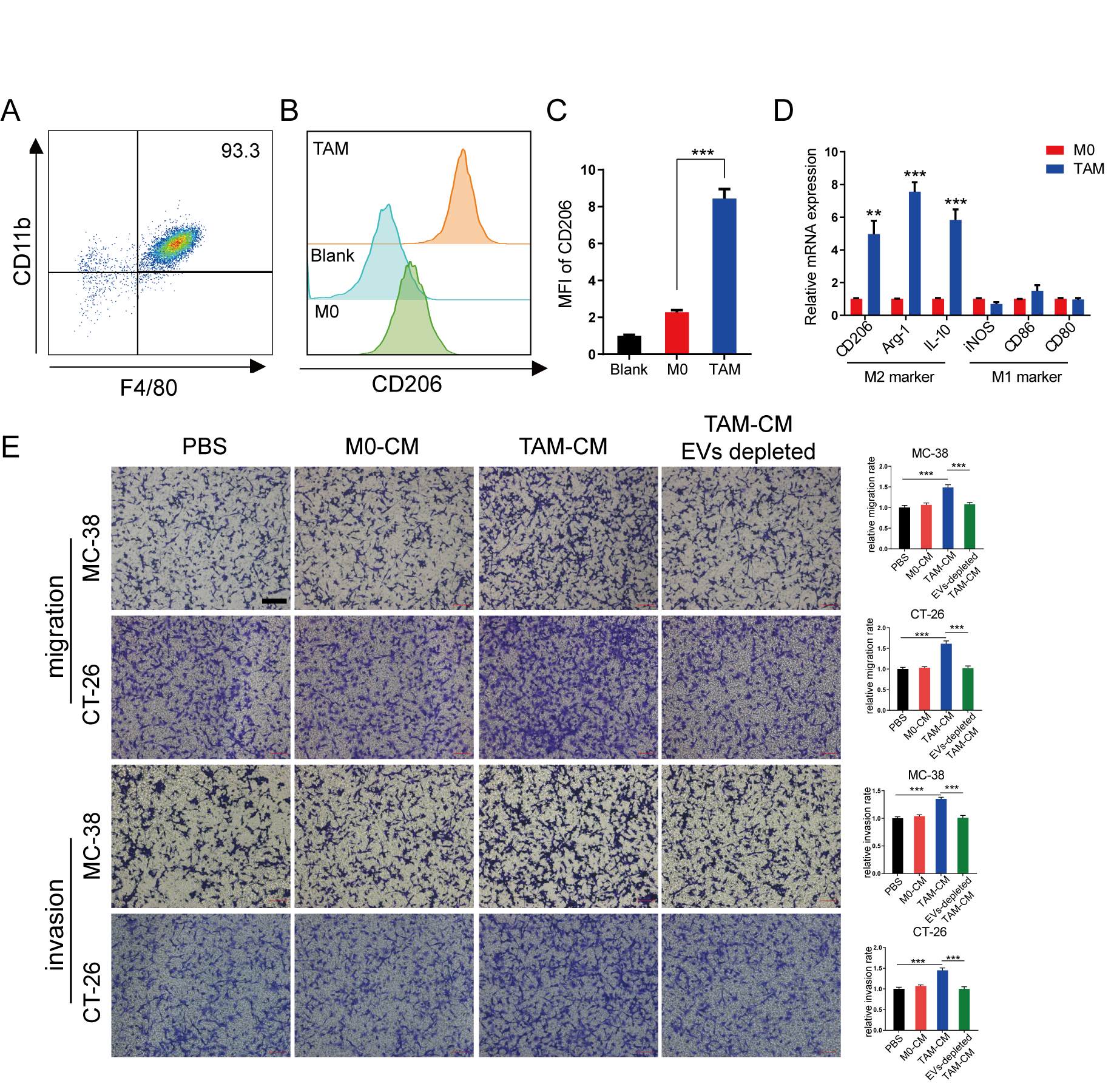

Supplement: Supplementary file 2 — Figure S2 TAMs promote the migration and invasion of CRC cells through Evs. (A–C) FACS analysis of the murine macrophage markers F4/80 and CD11b (A) and the TAM marker CD206 (B). The mean fluorescence intensity of CD206 is shown on the right (C). (D) qRT‒PCR was used to measure the levels of M2 markers (CD206, Arg‐1 and IL‐10) and M1 markers (INOS, CD86 and CD80). (E) Representative images of migration and invasion assays of CRC cells treated with EV‐depleted CM or untreated CM, accompanied by the quantification of migrated and invaded cells on the right. Scale bar: 200 μm. The data are presented as the means ± SDs. *p < .05, **p < .01, ***p < .001. [file CTM2-14-e1591-s001.tif]

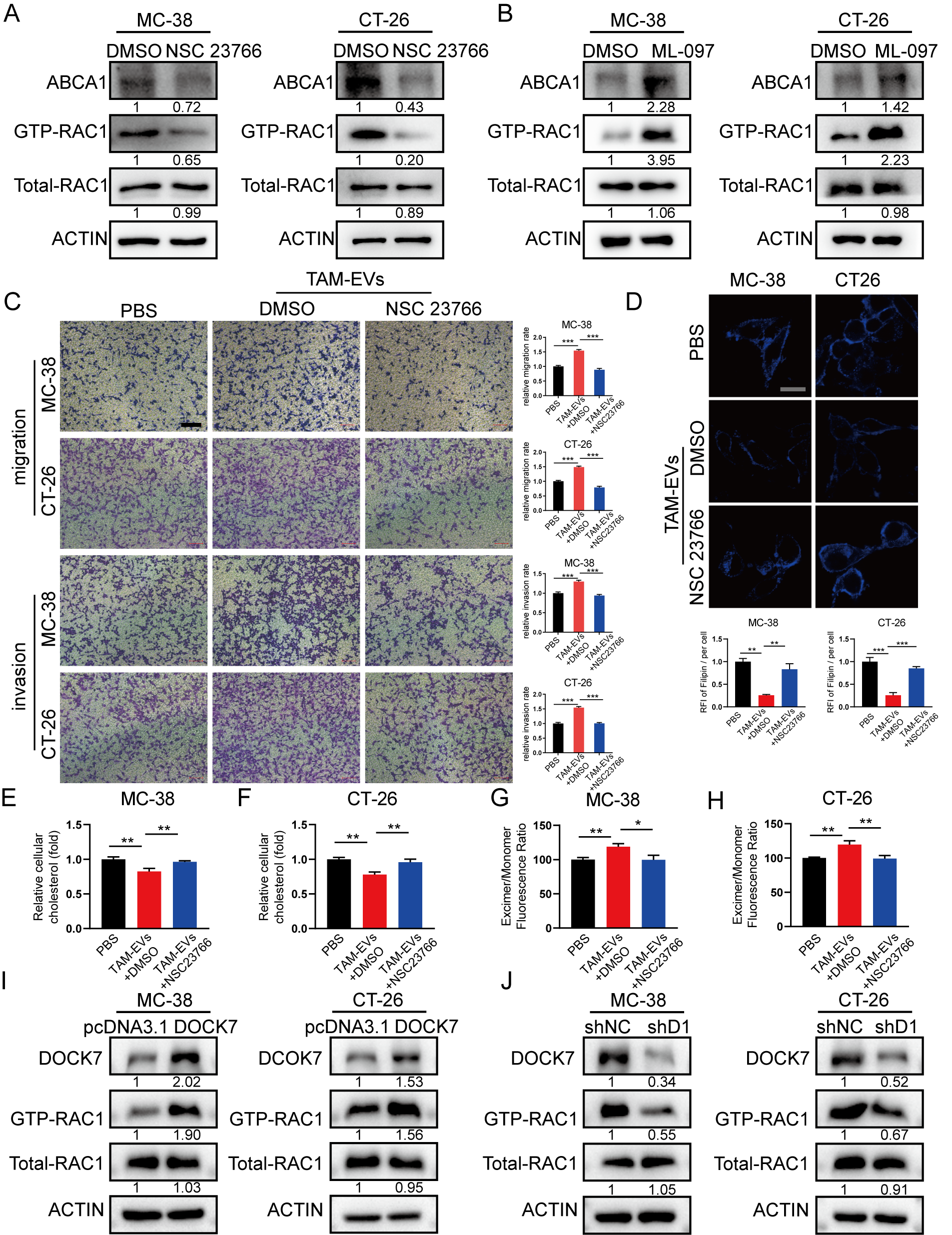

Supplement: Supplementary file 3 — Figure S3 TAM‐EVs regulate metastatic ability, cholesterol metabolism and membrane fluidity in CRC cells via activation of RAC1. (A and B) RAC1 activation and ABCA1 expression were evaluated by Western blot analysis in MC‐38 and CT‐26 cells treated with TAM‐EVs combined with NSC23766 or ML‐097. (C) Transwell assays were used to determine the effects of NSC23766 on the migration and invasion of CRC cells treated with TAM‐EVs, and the quantification of migrated and invaded cells is shown on the right. Scale bar: 200 μm. (D) Membrane cholesterol was evaluated by filipin staining in CRC cells treated with TAM‐EVs with or without NSC23766. Scale bar: 20 μm. The quantification of the mean fluorescence intensity (MFI) is shown at the bottom. (E and F) The total cholesterol content was measured in CRC cells treated with TAM‐EVs with or without NSC23766. (G and H) Membrane fluidity was evaluated in CRC cells treated with TAM‐EVs with or without NSC23766. (I and J) A GTP‐RAC1 pull‐down assay was performed to detect RAC1 activation in MC‐38 and CT‐26 cells with overexpression or silencing of DOCK7. The data are presented as the means ± SDs. *p < .05, **p < .01, ***p < .001. [file CTM2-14-e1591-s003.tif]

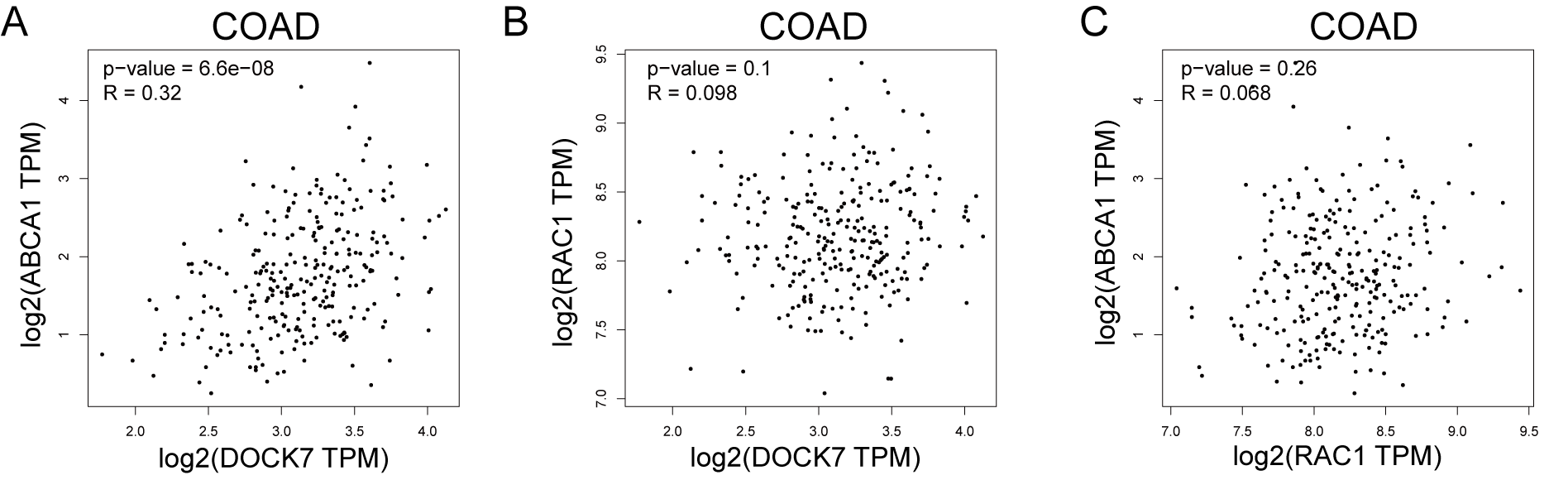

Supplement: Supplementary file 4 — Figure S4 Correlations among DOCK7, RAC1 and ABCA1 expression in CRC clinical samples. (A) A positive correlation was observed between DOCK7 and ABCA1 expression in CRC patients based on the GEPIA database. (B and C) No significant correlation was found between DOCK7 and RAC1 expression or between RAC1 and ABCA1 expression in CRC patients. [file CTM2-14-e1591-s006.tif]

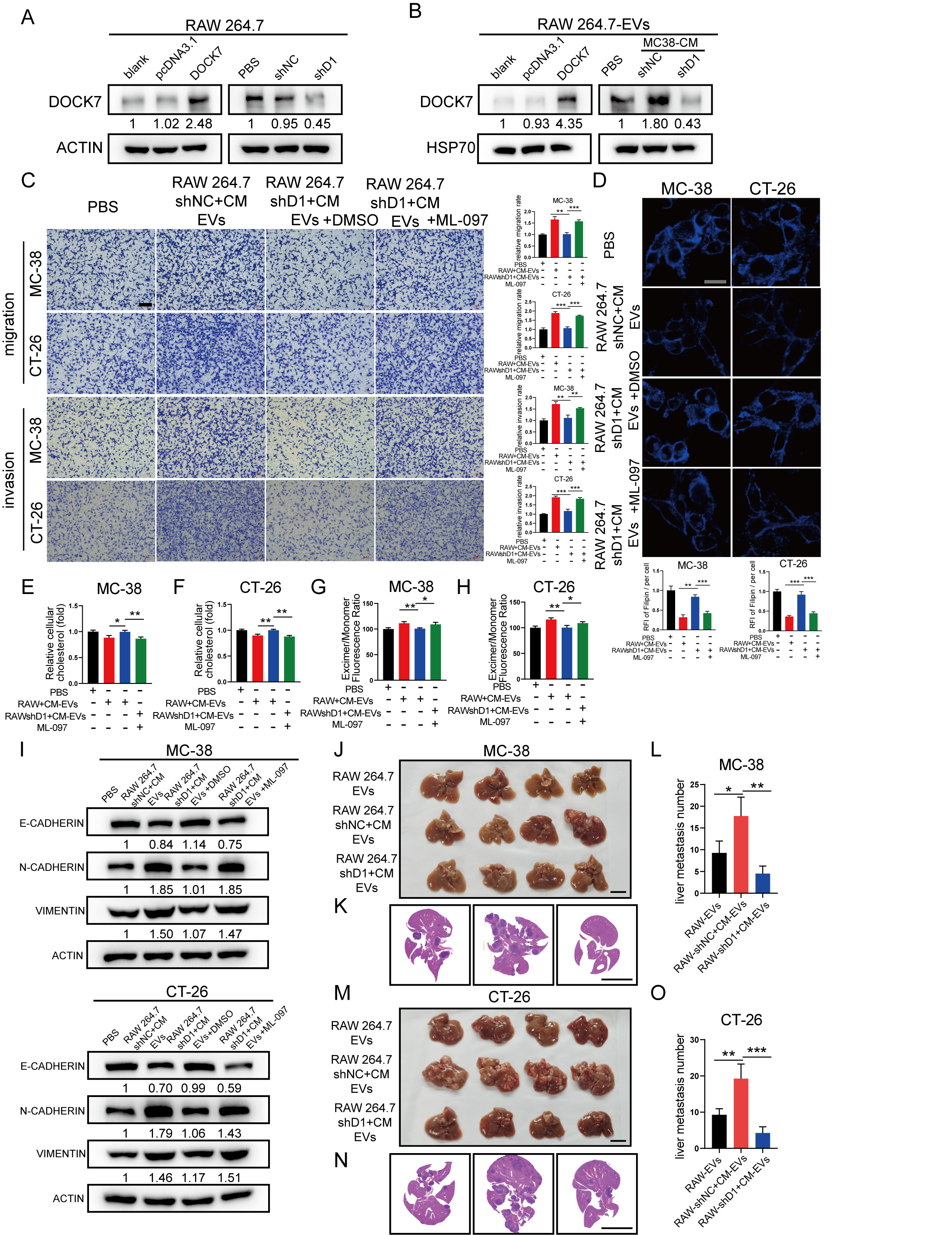

Supplement: Supplementary file 5 — Figure S5 DOCK7 in RAW264.7‐EVs could regulate the protumour phenotype mediated by ABCA1 via activation of RAC1. (A and B) Western blot analysis was used to evaluate the expression of DOCK7 in RAW264.7 cells transfected with pcDNA3.1‐DOCK7 or transduced with shRNA against DOCK7 (A) and in the corresponding RAW264.7‐EVs (B). (C–I) Migration and invasion assays (C), filipin staining (D), total cholesterol content measurement (E and F), a membrane fluidity assay (G and H) and Western blot analysis of EMT markers (I) were conducted to evaluate CRC cells treated with or without MC‐38 CM‐treated RAW264.7‐shNC/shDOCK7‐EVs alone or combined with ML‐097. Representative morphological (J and K) and HE staining images (L and M) of livers from a murine ectopic hepatic metastasis model established by intrasplenic injection of CRC cells treated with or without MC‐38 CM‐treated RAW264.7‐shNC/shDOCK7‐EVs. The quantification of liver metastatic nodules is shown at the bottom (N and O). Scale bar: 1 cm. The data are presented as the means ± SDs. *p < .05, **p < .01, ***p < .001. [file CTM2-14-e1591-s008.tif]

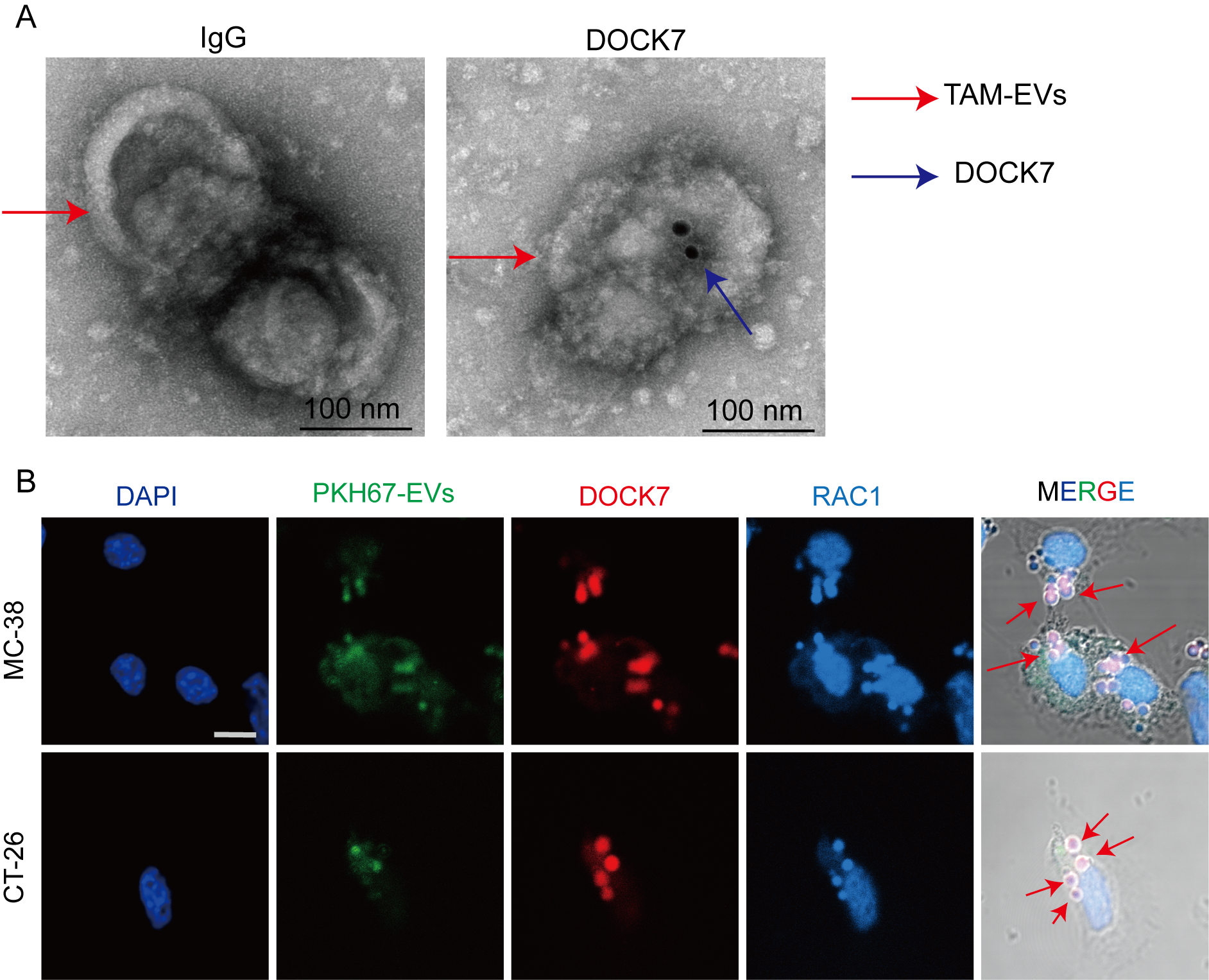

Supplement: Supplementary file 6 — Figure S6 DOCK7 packaged in EVs could bind to intracellular RAC1 in CRC cells. (A) Immunogold labelling electron microscopy showing the location of the DOCK7 protein in TAM‐EVs. Scale bar: 100 nm. (B) EV‐packaged DOCK7 was found to bind to intracellular RAC1 in CRC cells. Scale bar: 10 μm. [file CTM2-14-e1591-s005.tif]

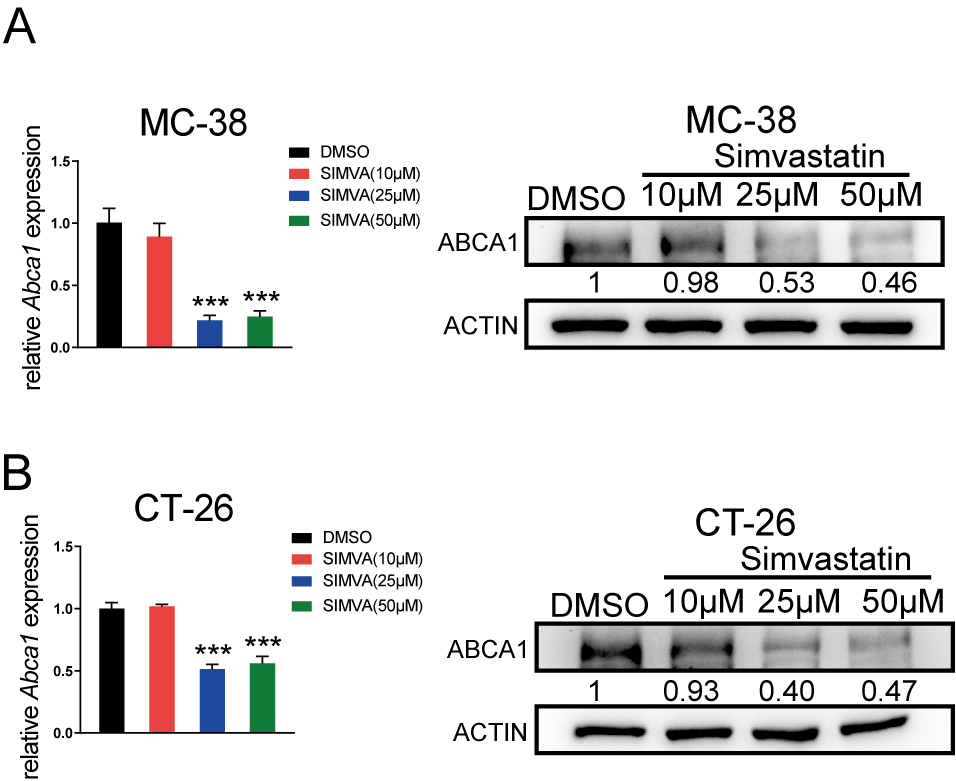

Supplement: Supplementary file 7 — Figure S7 Simvastatin decreased the mRNA and protein levels of ABCA1 in CRC cells. (A and B) qRT‒PCR and Western blot analyses were conducted to measure the mRNA and protein expression levels of ABCA1 in MC‐38 (A) and CT‐26 (B) cells treated with simvastatin. The data are presented as the means ± SDs. *p < .05, **p < .01, ***p < .001. [file CTM2-14-e1591-s004.tif]

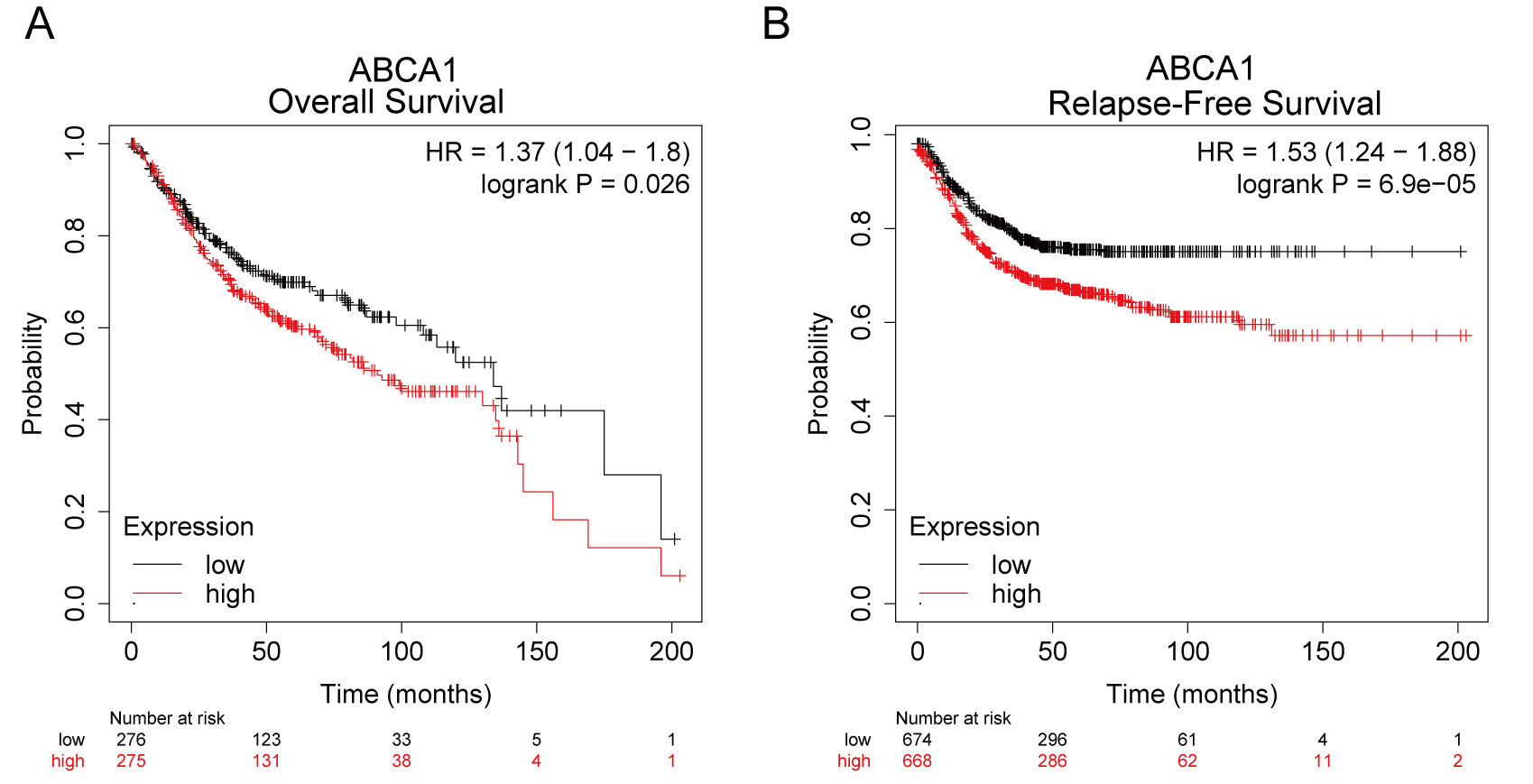

Supplement: Supplementary file 8 — Figure S8 ABCA1 expression is positively correlated with poor prognosis in CRC patients. (A and B) The expression level of ABCA1 was positively correlated with poor overall survival (A) and relapse‐free survival (B) according to the Kaplan‒Meier Plotter database. [file CTM2-14-e1591-s010.tif]
