# Supplementary material for: Tumour‐associated macrophage‐derived DOCK7‐enriched extracellular vesicles drive tumour metastasis in colorectal cancer via the RAC1/ABCA1 axis
Source: Clin Transl Med. 2024 Feb 22;14(2):e1591. doi: 10.1002/ctm2.1591 (PMC10883245; doi:10.1002/ctm2.1591)
Supplement: Supplementary file 9 — Supporting Table S1 cDNA target sequences of the shRNAs. Supporting Table S2 Primary antibodies used for Western blot analysis. Supporting Table S3 Primer sequences used for real‐time PCR. [file CTM2-14-e1591-s007.docx]

Supplemental Tables

Supplemental Table 1.

cDNA target sequences of the shRNAs.

| Name | Species | Sequence 5’——3’ |
| --- | --- | --- |
| shABCA1-1 | Mouse | CCAAAGAGATACCAGCATT |
| shABCA1-2 | Mouse | TGGGTGTCAGTAATTCTCA |
| shABCA1-3 | Mouse | ACGAGGATAACAACTACAA |
| shDOCK7-1 | Mouse | CGGTATATTCTTTAATGAT |
| shDOCK7-2 | Mouse | CATTTATAGGTCCATTCAT |

Supplemental Table 2.

Primary antibodies used for Western blot analysis.

| Antibody | Company name | Dilution | Catalog number |
| --- | --- | --- | --- |
| CD9 | ABclonal | 1:1000 | A1703 |
| CD63 | ABclonal | 1:1000 | A5271 |
| ALIX | Proteintech | 1:1000 | 12422-1-AP |
| TSG101 | Proteintech | 1:1000 | 28283-1-AP |
| HSP70 | ABclonal | 1:1000 | A12948 |
| E-CADHERIN | ABclonal | 1:1000 | A22333 |
| N-CADHERIN | Cell Signaling Technology | 1:500 | 13116S |
| VIMENTIN | Arigo | 1:1000 | ARG66302 |
| β-Actin | MBL | 1:5000 | PM053-7 |
| ABCA1 | Protintech | 1:1000 | 26564-1-AP |
| ABCA1 | Abcam | 1:1000 | Ab18180 |
| DOCK7 | Protintech | 1:1000 | 13000-1-AP |
| RAC1 | cytoskeleton | 1:500 | ARC03 |
| p-AKT | ABclonal | 1:1000 | AP0637 |
| AKT | ABclonal | 1:1000 | A17909 |
| p-FOXO1 | ABclonal | 1:1000 | AP0172 |
| FOXO1 | ABclonal | 1:1000 | A2934 |
| Anti-rabbit IgG | Cell Signaling Technology | 1:5000 | #7074 |
| Anti-mouse IgG | Cell Signaling Technology | 1:5000 | #7076 |

Supplemental Table 3.

Primer sequences used for real-time PCR.

| Name | Species |  | Sequence 5’——3’ |
| --- | --- | --- | --- |
| β-Actin | Mouse | Forward | TCCTCCCTGGAGAAGAGCTA |
|  |  | Reverse | ATCTCCTTCTGCATCCTGTC |
| GAPDH | Mouse | Forward | ACTGAGGACCAGGTTGTC |
|  |  | Reverse | TGCTGTAGCCGTATTCATTG |
| MRC1 | Mouse | Forward | AGTCAGAACAGACTGCGTGG |
|  |  | Reverse | CCAGAGGGATCGCCTGTTTT |
| ARG1 | Mouse | Forward | CTTGCGAGACGTAGACCCTG |
|  |  | Reverse | TGAGTTCCGAAGCAAGCCAA |
| IL-10 | Mouse | Forward | ACTTGGGTTGCCAAGCCTTA |
|  |  | Reverse | GACACCTTGGTCTTGGAGCTTA |
| NOS2 | Mouse | Forward | AACAGGGAGAAAGCGCAAAA |
|  |  | Reverse | CCTCACATACTGTGGACGGG |
| CD86 | Mouse | Forward | CTTACGGAAGCACCCACGAT |
|  |  | Reverse | TGTAAATGGGCACGGCAGAT |
| CD80 | Mouse | Forward | ACTAGTTTCTCTTTTTCAGGTTGTG |
|  |  | Reverse | GAGCCAATGGAGCTTAGGCA |
| DOCK7 | Mouse | Forward | TTGTCGCCGAGTATCTGAGC |
|  |  | Reverse | CCAGTGCTCTGATGGACGATT |
| APOC1 | Mouse | Forward | ACAGCTAAGGGATTGGGGTG |
|  |  | Reverse | ACGACCACAATCAGGACAGG |
| APOB | Mouse | Forward | AGCCAATAATGTGAGCCCCT |
|  |  | Reverse | TCATCTTGAGTTCAGGCTGCTT |
| ABCA1 | Mouse | Forward | GGGCTCCTCCCTGTTTTTGA |
|  |  | Reverse | GTCAGCGTGTCACTTTCATGG |
| APOH | Mouse | Forward | AGCCGGATGACCTACCATTTG |
|  |  | Reverse | ATCTCAGGGTGTTGATGGGC |
| APOA4 | Mouse | Forward | TACGTATGCTGATGGGGTGC |
|  |  | Reverse | TCTGCATGTTCTCCCCGAAC |
| RPL13A | Mouse | Forward | GAGGTCGGGTGGAAGTACCA |
|  |  | Reverse | TGCATCTTGGCCTTTTCCTT |
